# Supplementary material for: A Saprophytic Fungus Tubeufia rubra Produces Novel Rubracin D and E Reversing Multidrug Resistance in Cancer Cells
Source: J Fungi (Basel). 2023 Feb 28;9(3):309. doi: 10.3390/jof9030309 (PMC10058889; doi:10.3390/jof9030309)
Supplement: Supplementary file 1 [file jof-09-00309-s001.zip › jof-2160217-supplementary.pdf]

A Saprobic Fungus Tubeufia rubra produces Novel Glyceroglycolipids viz Rubracin D and E Reversing Multidrug Resistance in Cancer Cells

Shengyan Qian[1,2,3], Xuebo Zeng[1,2], Yixin Qian[1], Yongzhong Lu[1,4], Zhangjiang He[1*], Jichuan Kang[1,2*]

[1*]Engineering and Research Center for Southwest Bio-Pharmaceutical Resources of National Education Ministry of China, Guizhou University, Guiyang 550025, P.R. China
[2] School of Life Science, Guizhou University, Guiyang 550025, P. R. China
[3]Guizhou Key Laboratory of Characteristic Microbial Research & Drug Development, Zunyi Medical University, Zunyi 563000, P. R. China
[4]School of Food and Pharmaceutical Engineering, Guizhou Institute of Technology, Guiyang 550003, P. R. China

Contents

S1: ORD spectrum of compound **1** 4
S2: HRESIMS spectrum of compound **1** 5
S3: UV spectrum of compound **1** 6
S4: IR spectrum of compound **1** 7
S5: ^{1}H NMR spectrum of compound **1** 8
S6: ^{1}H NMR spectrum of part expansion compound **1** (methanol-d_4) 9
S7: ^{13}C NMR and DEPT spectra of compound **1** 10
S8: HSQC spectrum of compound **1** 11
S9: HMBC spectrum of compound **1** 12
S10: COSY spectrum of compound **1** 13
S11: HRESIMS spectrum of compound **2** 14
S12: ORD spectrum of compound **2** 15
S13: UV spectrum of compound **2** 16
S14: IR spectrum of compound **2** 17
S15: ^{1}H NMR spectrum of compound **2** 18
S16: ^{1}H NMR spectrum of part expansion compound **2** (methanol-d_4) 19
S17: ^{13}C NMR and DEPT spectra of compound **2** 20
S18: HSQC spectrum of compound **2** 21
S19: HMBC spectrum of compound **2** 22
S20: COSY spectrum of compound **2** 23

S21: Cytotoxicity raw data of **1** and **2** against MCF-7/ADM 24
S22: Cytotoxicity raw data of ADM against MCF-7/ADM 25
S23: Reversed MDR activity raw data of **1** MCF-7/ADM........ 26
S24: Reversed MDR activity raw data of **2** MCF-7/ADM........ 27
S25: Reversed MDR activity raw data of Vrp MCF-7/ADM........ 28
S26: Cytotoxicity raw data of **1** and **2** against A549/ADM........ 29
S27: Cytotoxicity raw data of ADM against A549/ADM 30
S28: Reversed MDR activity raw data of **1** A549/ADM........ 31
S29: Reversed MDR activity raw data of **2** A549/ADM........ 32
S30: Reversed MDR activity raw data of Vrp A549/ADM........ 33
S31: Cytotoxicity raw data of **1** and **2** against K562/ADM 34
S32: Cytotoxicity raw data of ADM against K562/ADM........ 35
S33: Reversed MDR activity raw data of **1** K562/ADM 36
S34: Reversed MDR activity raw data of **2** K562/ADM 37
S35: Reversed MDR activity raw data of Vrp K562/ADM........ 38
Spectroscopic data of known compounds **3**–1**8** 39-46
The UHPLC-ESI-MS analysis of the crude ethyl acetate extract of PF02-2 and oat medium 47-51

Rudolph Research Analytical

This sample was measured on an Autopol VI, Serial #91058
Manufactured by Rudolph Research Analytical, Hackettstown, NJ, USA.

Measurement Date : Monday, 12-JUL-2021

Set Temperature : OFF

Time Delay : Disabled

Delay between Measurement : Disabled

n	Average	Std.Dev.	% RSD	Maximum	Minimum
5	62.06	0.35	0.56	62.46	61.62

S.No	Sample ID	Time	Result	Scale	OR °Arc	WLG.nm	Lg.mm	Conc.g/100ml	Temp.
1	PF70	04:24:20 PM	61.62	SR	0.0801	589	100.00	0.130	28.8
2	PF70	04:24:29 PM	61.77	SR	0.0803	589	100.00	0.130	28.8
3	PF70	04:24:37 PM	62.23	SR	0.0809	589	100.00	0.130	28.8
4	PF70	04:24:45 PM	62.23	SR	0.0809	589	100.00	0.130	28.8
5	PF70	04:24:53 PM	62.46	SR	0.0812	589	100.00	0.130	28.8

Figure S1 ORD spectrum of compound **1**

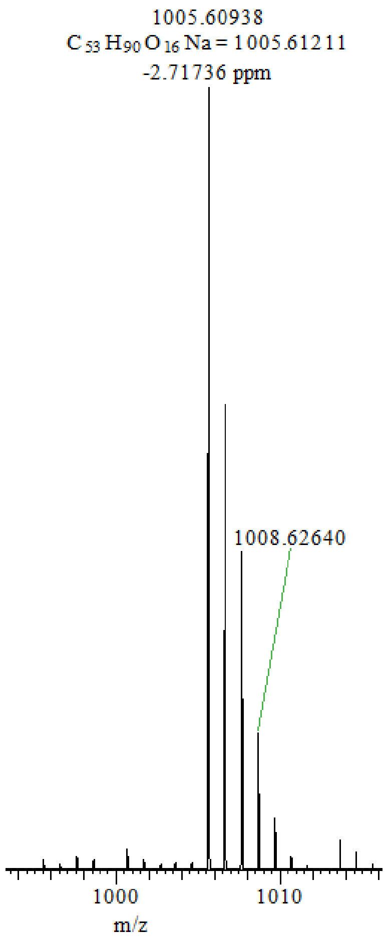

Figure S2 HRESIMS spectrum of compound **1**

数据集: PF70 - RawData

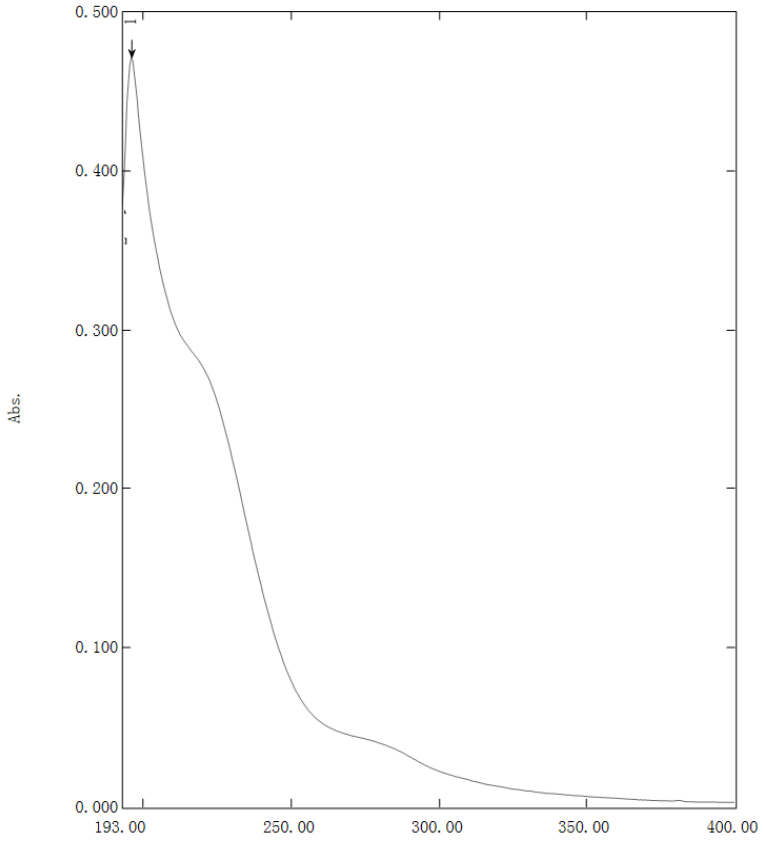

Figure S3 UV spectrum of compound **1**

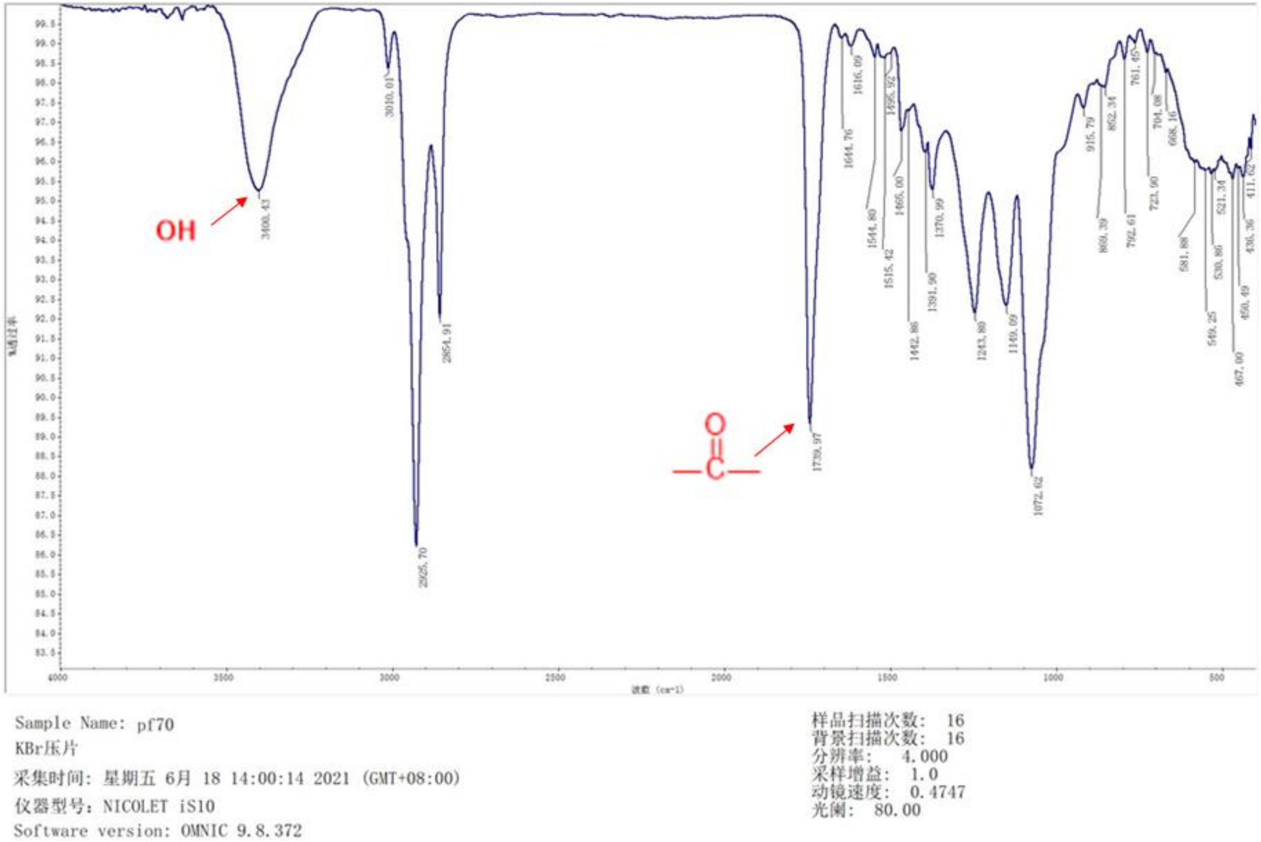

Figure S4 IR spectrum of compound **1**

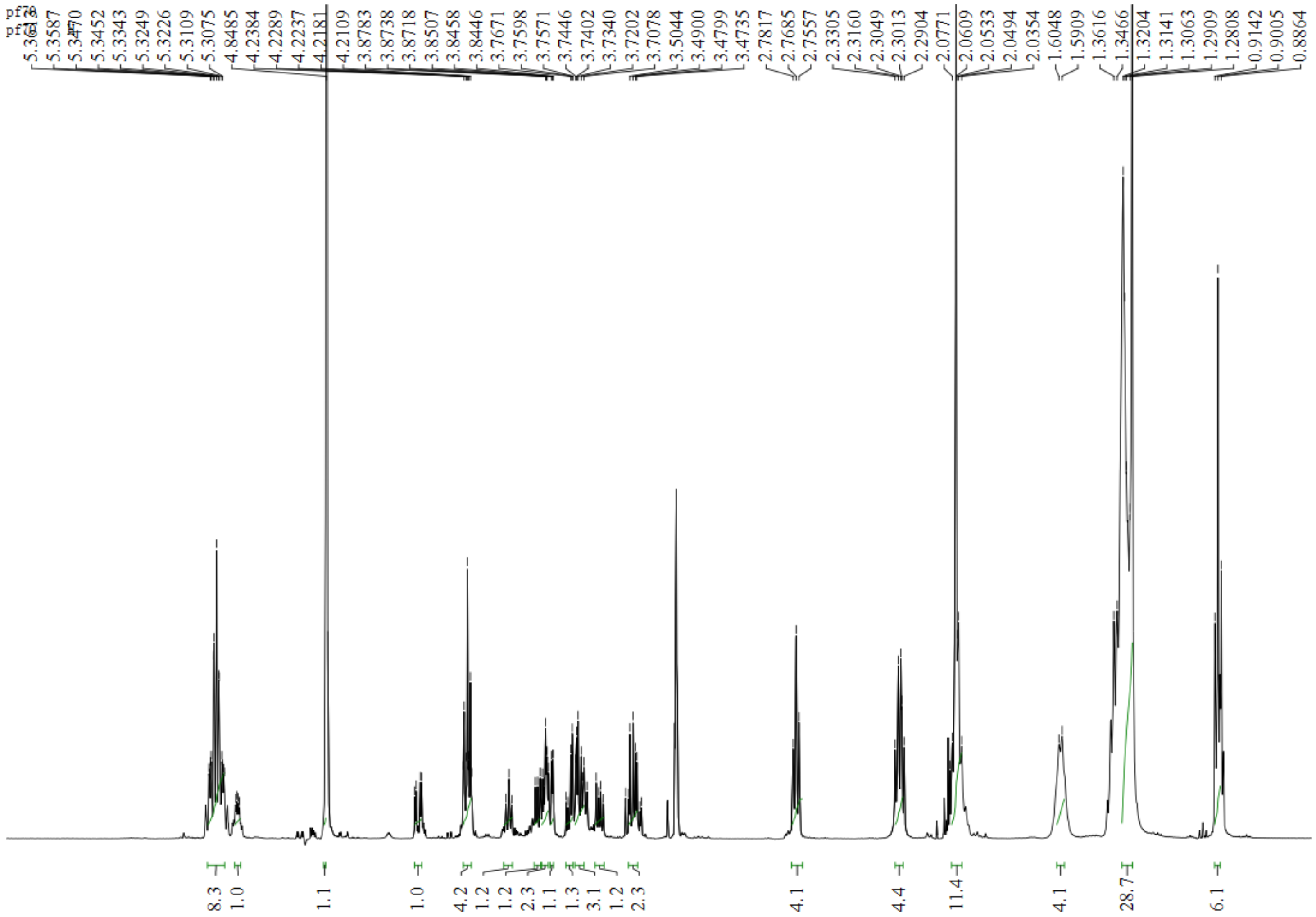

Figure S5 ^{1}H NMR spectrum of compound **1**

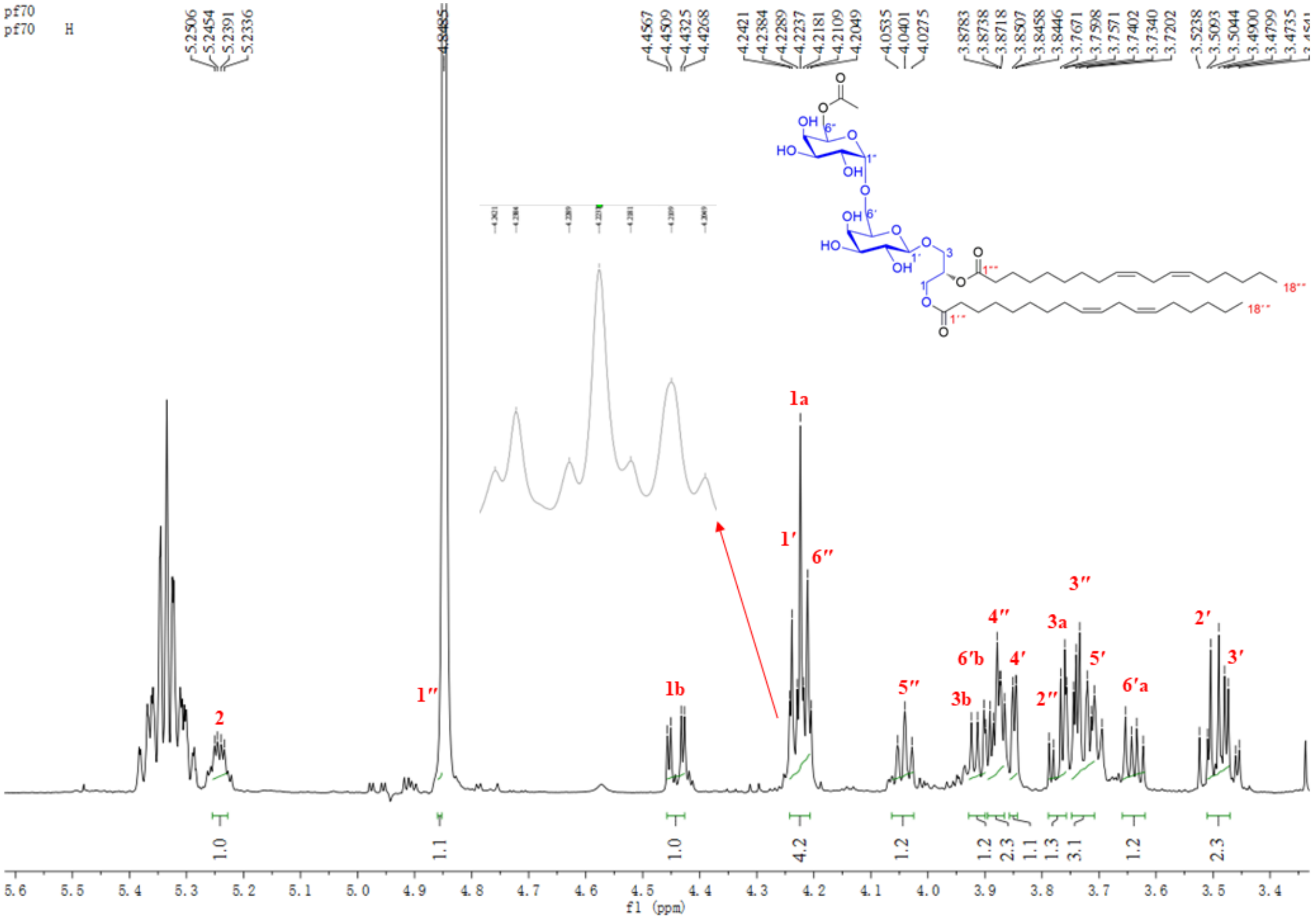

Figure S6 ^{1}H NMR spectrum of part expansion from compound **1** (methanol -*d*4)

In the extensive ^{1}H-NMR spectrum of compound **1**, the signal δ_H at 4.85 coincides with the solvent peak of methanol-*d*4, but the combination of HSQC and HMBC spectrum can imply that the signal at δ_H at 4.85 is H-1″ of *α*-*D*-galactopyranose. Similarly, the H-1′ signal of β-D-galactopyranose overlaps partially with H-1a and H-6", but the H-1' coupling constant can be calculated according to the splitting law of coupling constant.

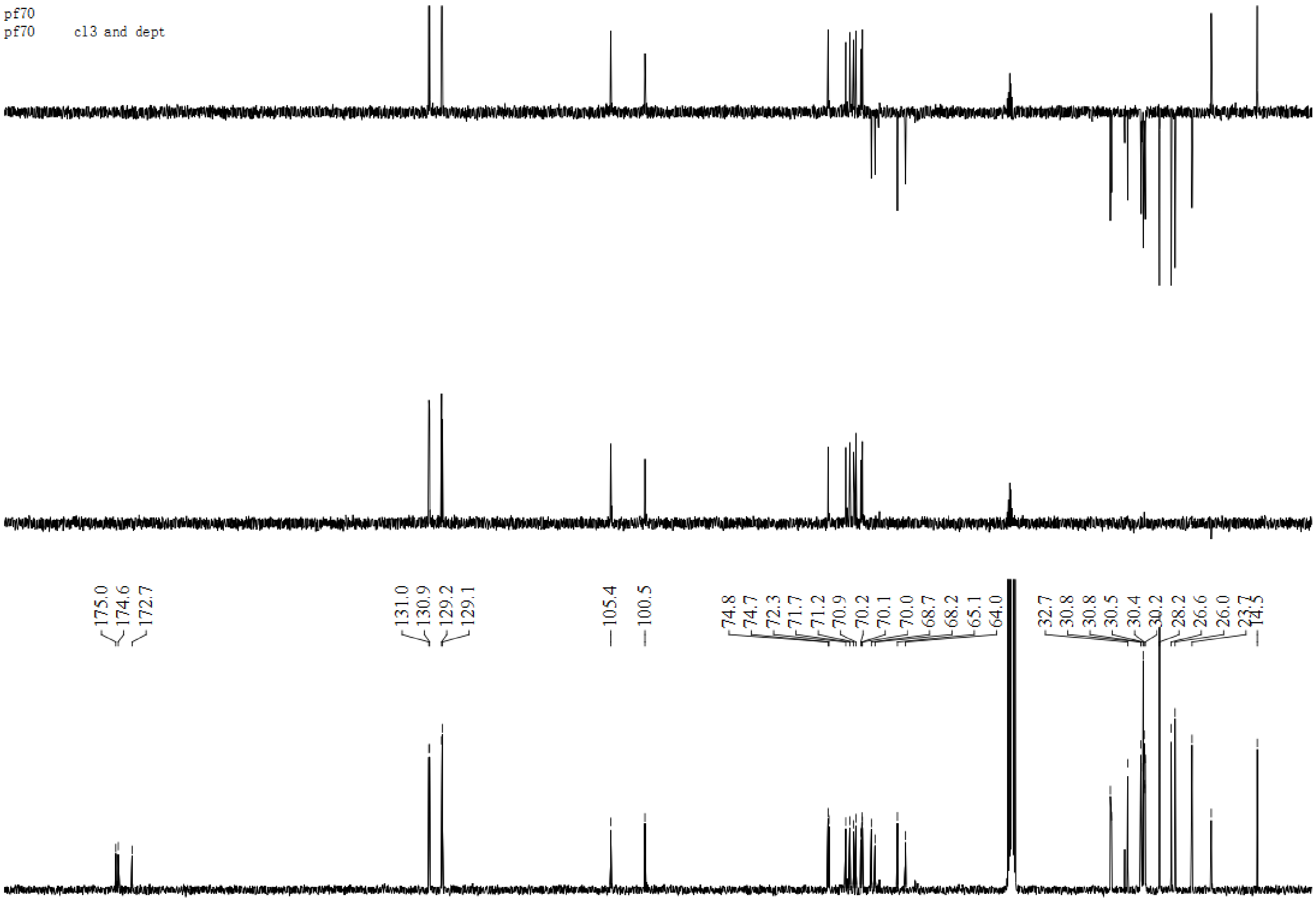

Figure S7 ^{13}C NMR and DEPT spectra of compound **1**

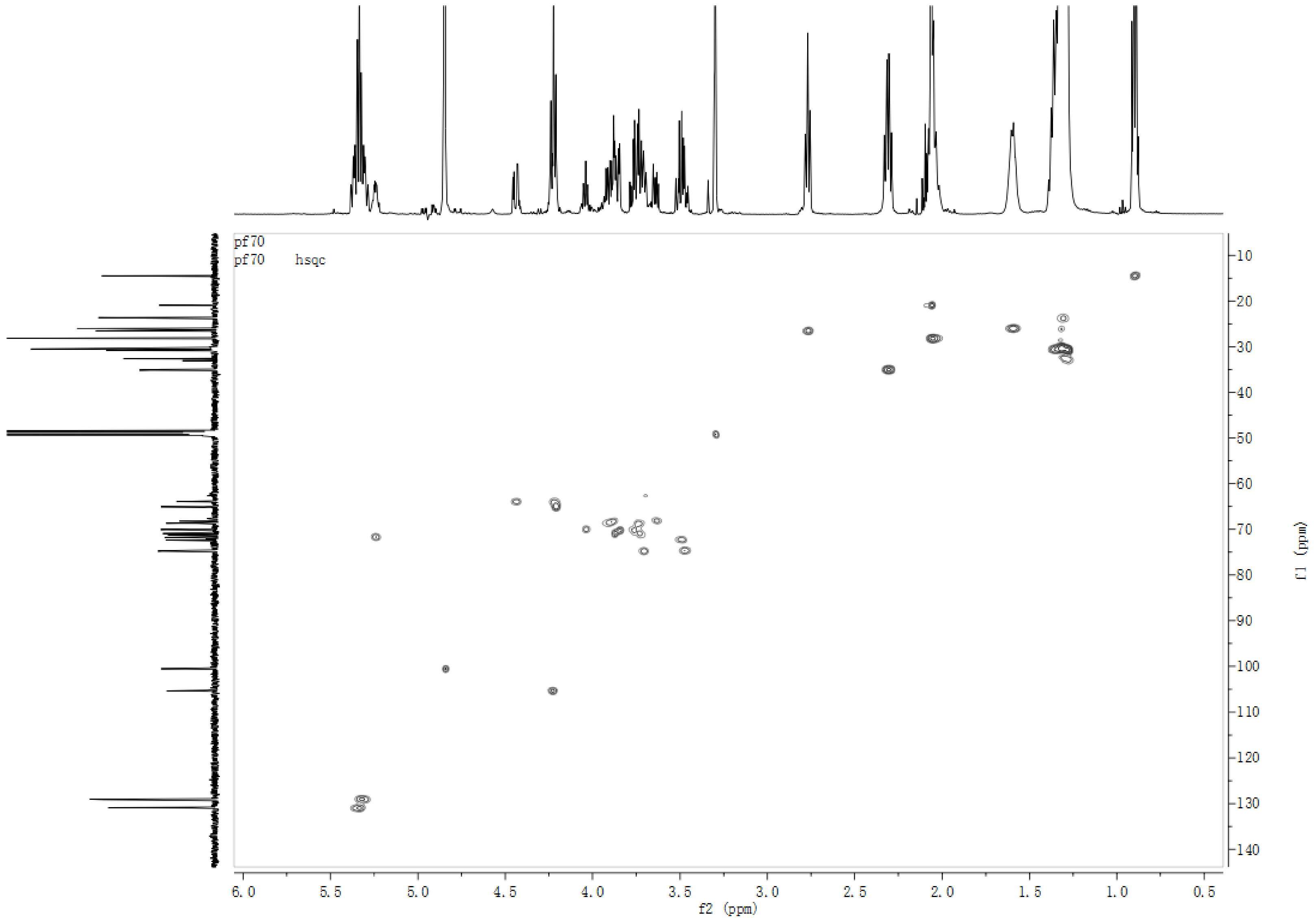

Figure S8 HSQC spectrum of compound **1**

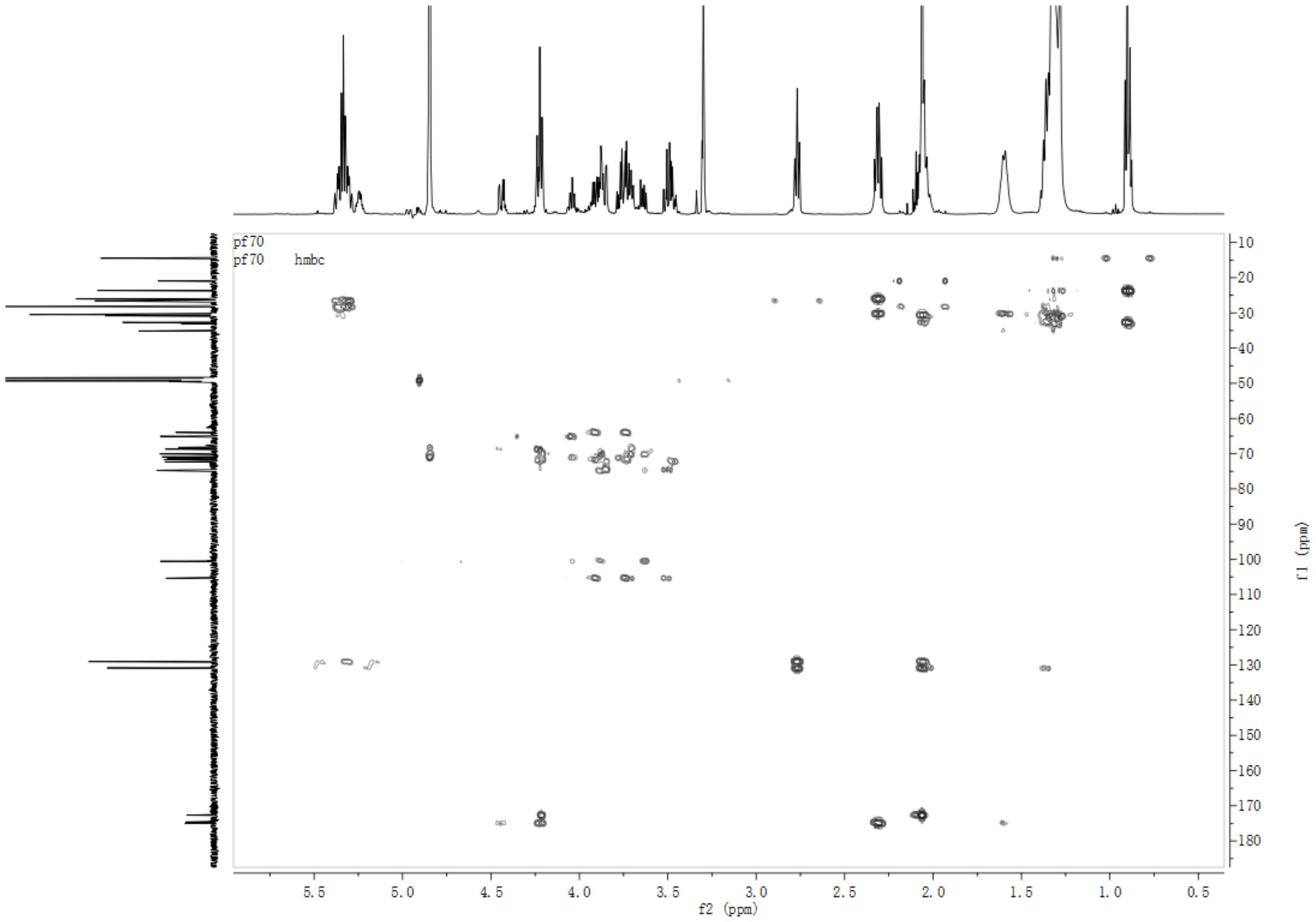

Figure S9 HMBC spectrum of compound **1**

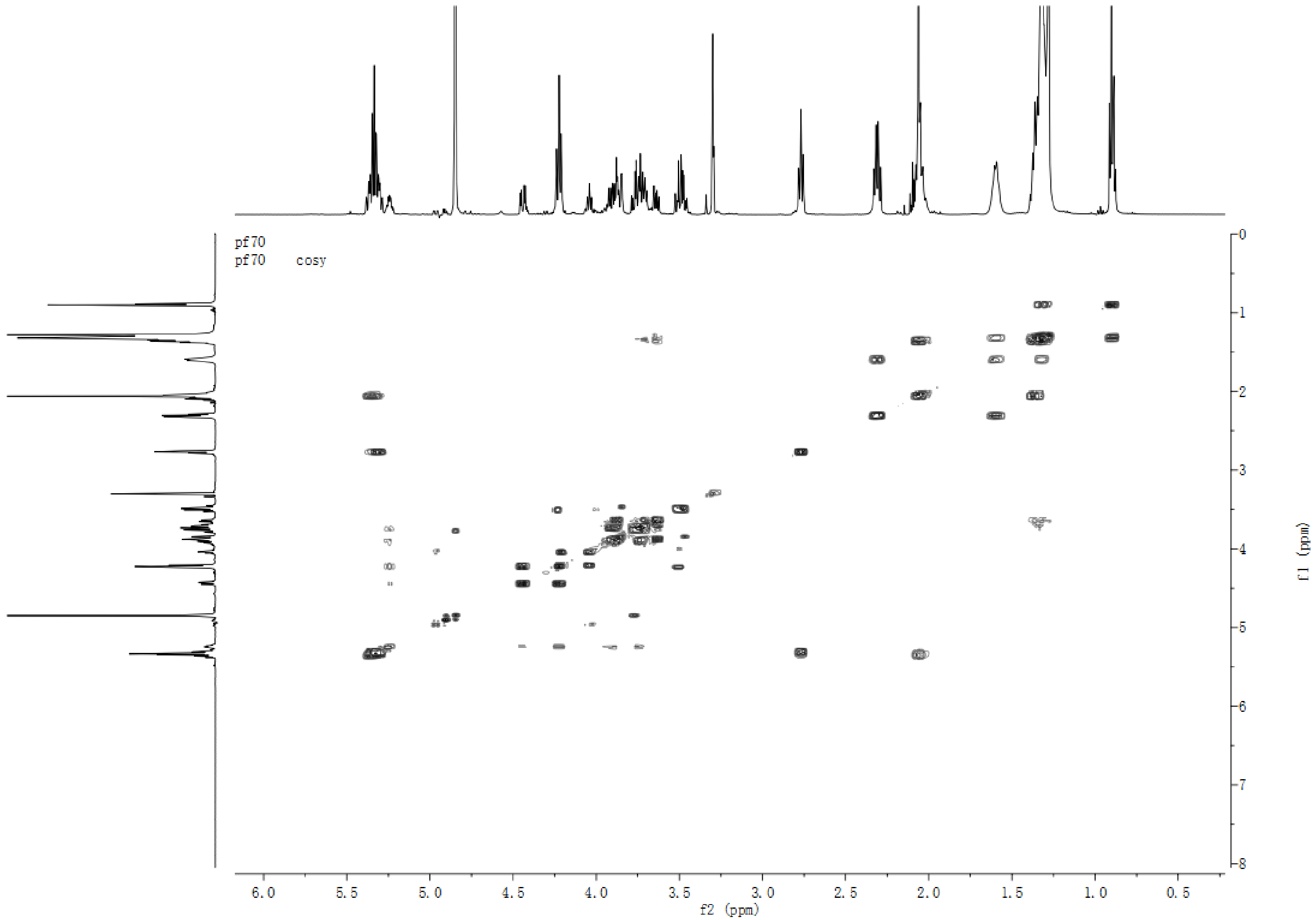

Figure S10 ^{1}H-^{1}H COSY spectrum of compound **1**

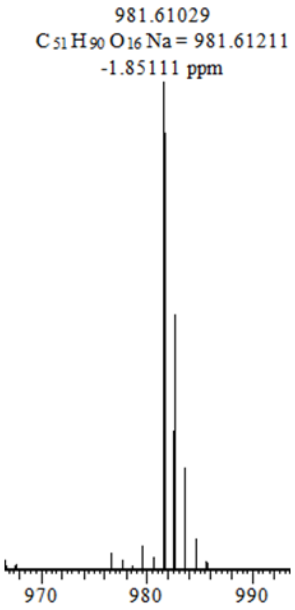

Figure S11 HRESIMS spectrum of compound **2**

Rudolph Research Analytical

This sample was measured on an Autopol VI, Serial #91058
Manufactured by Rudolph Research Analytical, Hackettstown, NJ, USA.

Measurement Date : Monday, 12-JUL-2021

Set Temperature : OFF

Time Delay : Disabled

Delay between Measurement : Disabled

n	Average	Std.Dev.	% RSD	Maximum	Minimum
5	73.85	0.45	0.60	74.45	73.27

S.No	Sample ID	Time	Result	Scale	OR °Arc	WLG.nm	Lg.mm	Conc.g/100ml	Temp.
1	PF52	04:17:53 PM	73.27	SR	0.0806	589	100.00	0.110	28.8
2	PF52	04:18:02 PM	73.64	SR	0.0810	589	100.00	0.110	28.8
3	PF52	04:18:10 PM	73.82	SR	0.0812	589	100.00	0.110	28.8
4	PF52	04:18:18 PM	74.09	SR	0.0815	589	100.00	0.110	28.8
5	PF52	04:18:26 PM	74.45	SR	0.0819	589	100.00	0.110	28.8

Figure S12 ORD spectrum of compound **2**

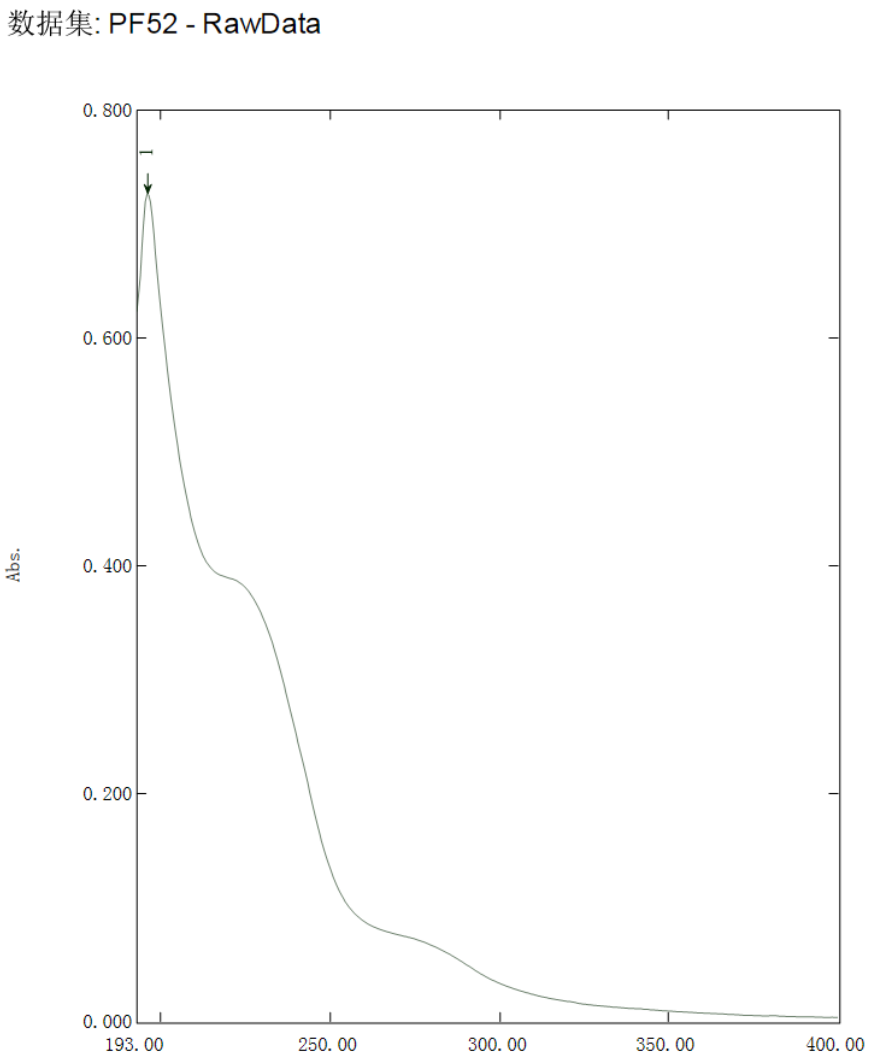

Figure S13 UV spectrum of compound **2**

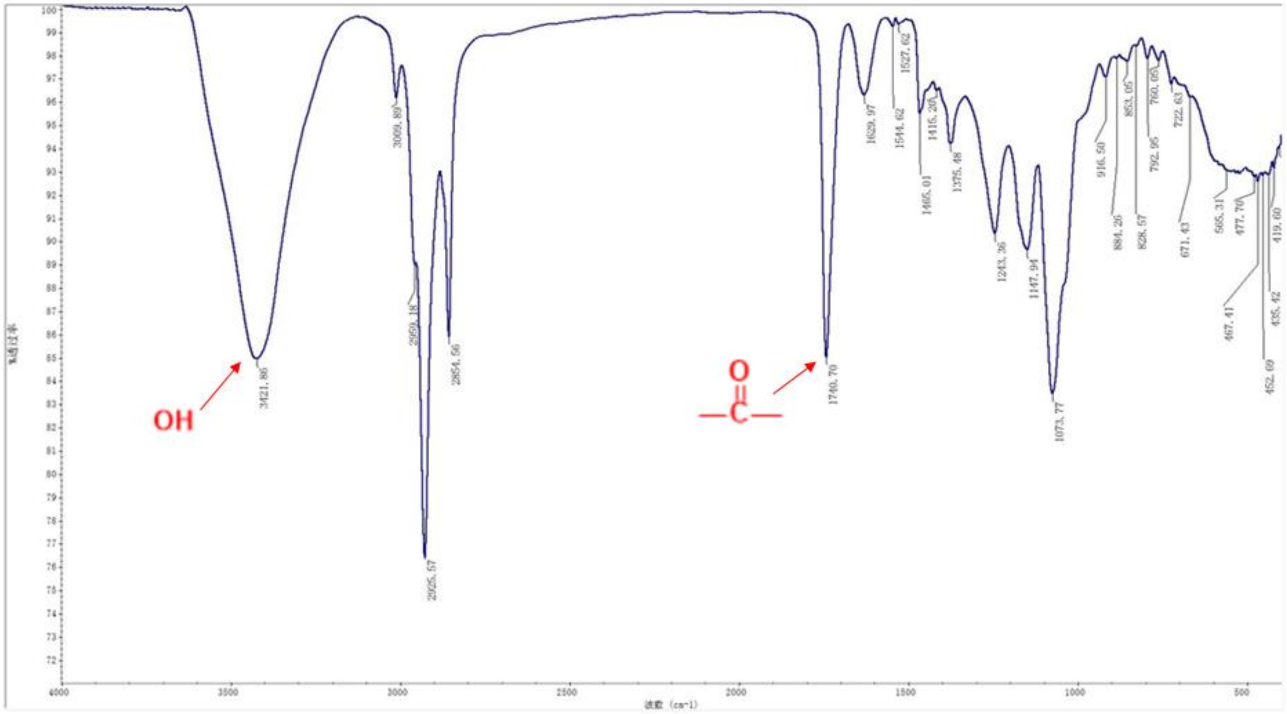

Sample Name: pf52

KBr压片

采集时间: 星期五 6月 18 11:08:26 2021 (GMT+08:00)

仪器型号: NICOLET iS10

Software version: OMNIC 9.8.372

样品扫描次数: 16

背景扫描次数: 16

分辨率: 4.000

采样增益: 1.0

动镜速度: 0.4747

光阑: 80.00

Figure S14 IR spectrum of compound **2**

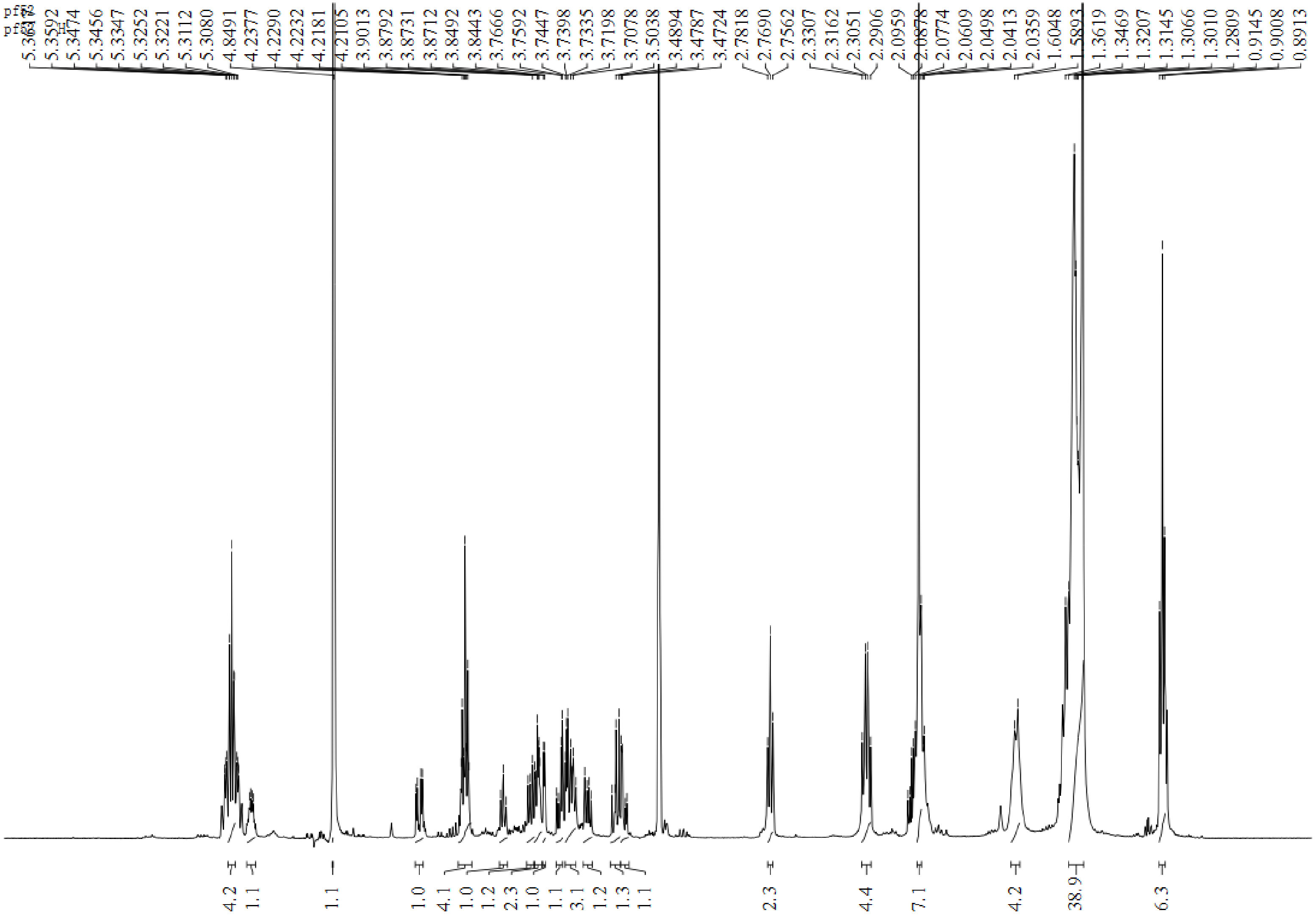

Figure S15 ^{1}H NMR spectrum of compound **2**

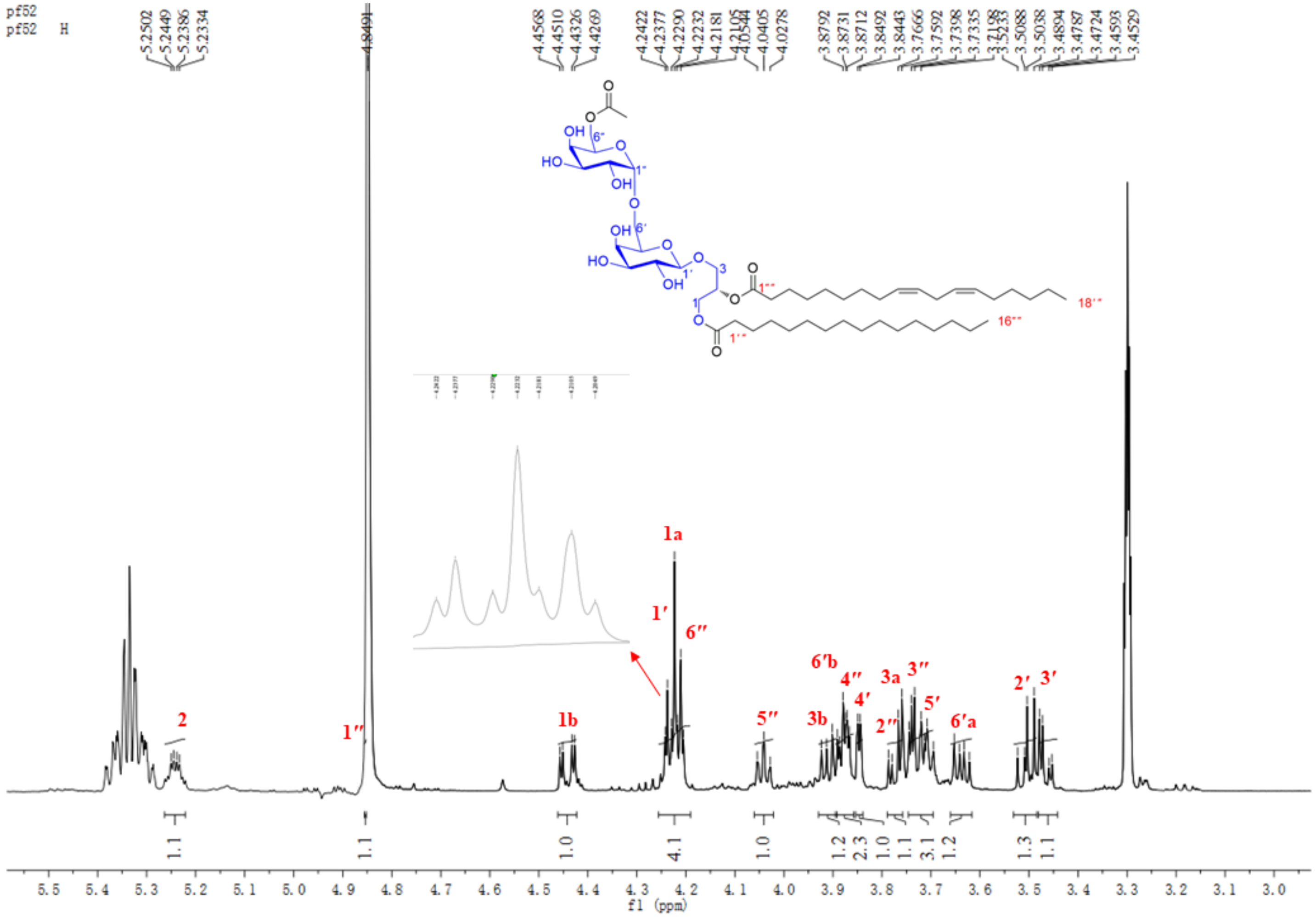

Figure S16 ^{1}H NMR spectrum of part expansion from compound **2** (methanol -d_4)

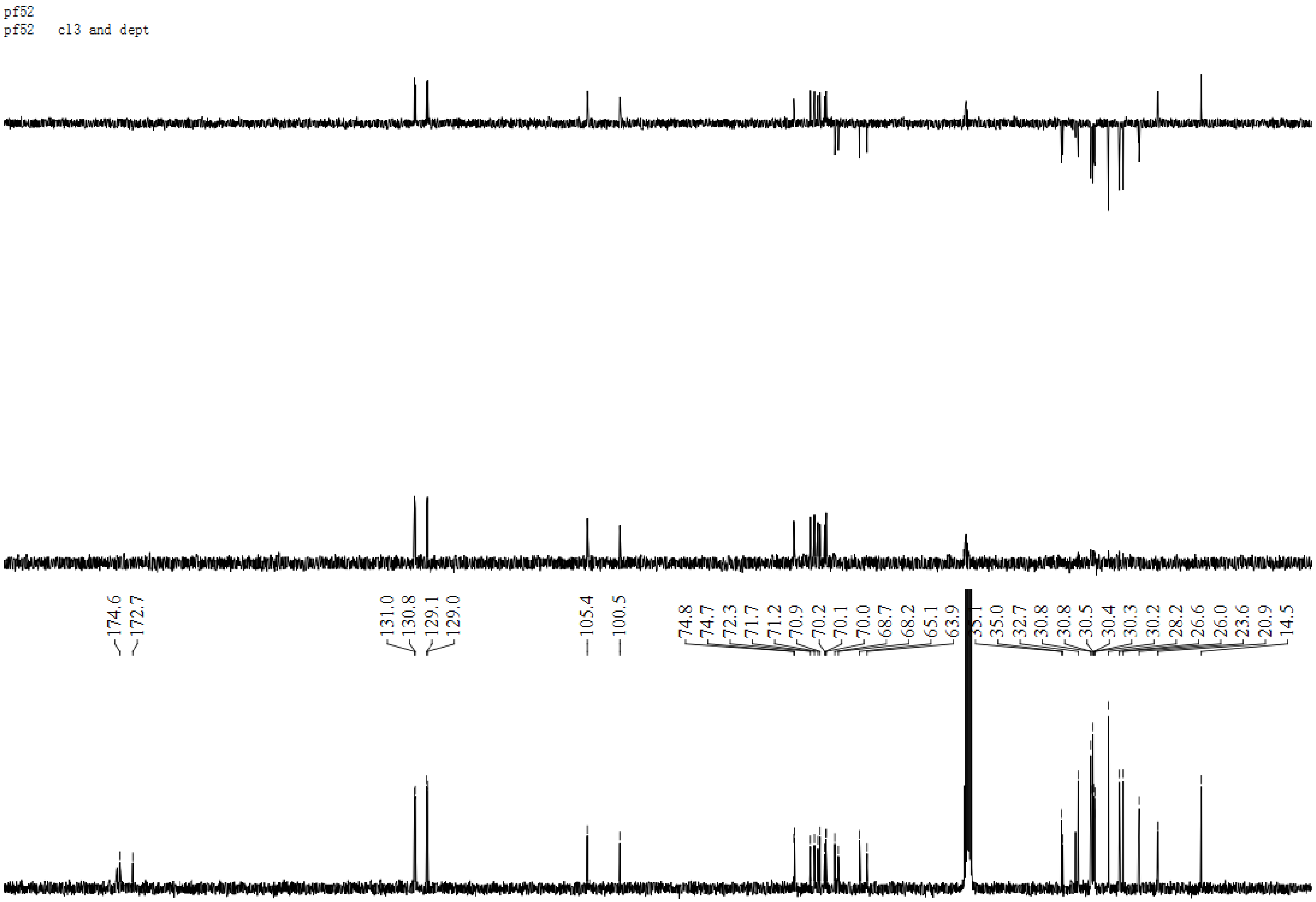

Figure S17 ^{13}C NMR and DEPT spectra of compound **2**

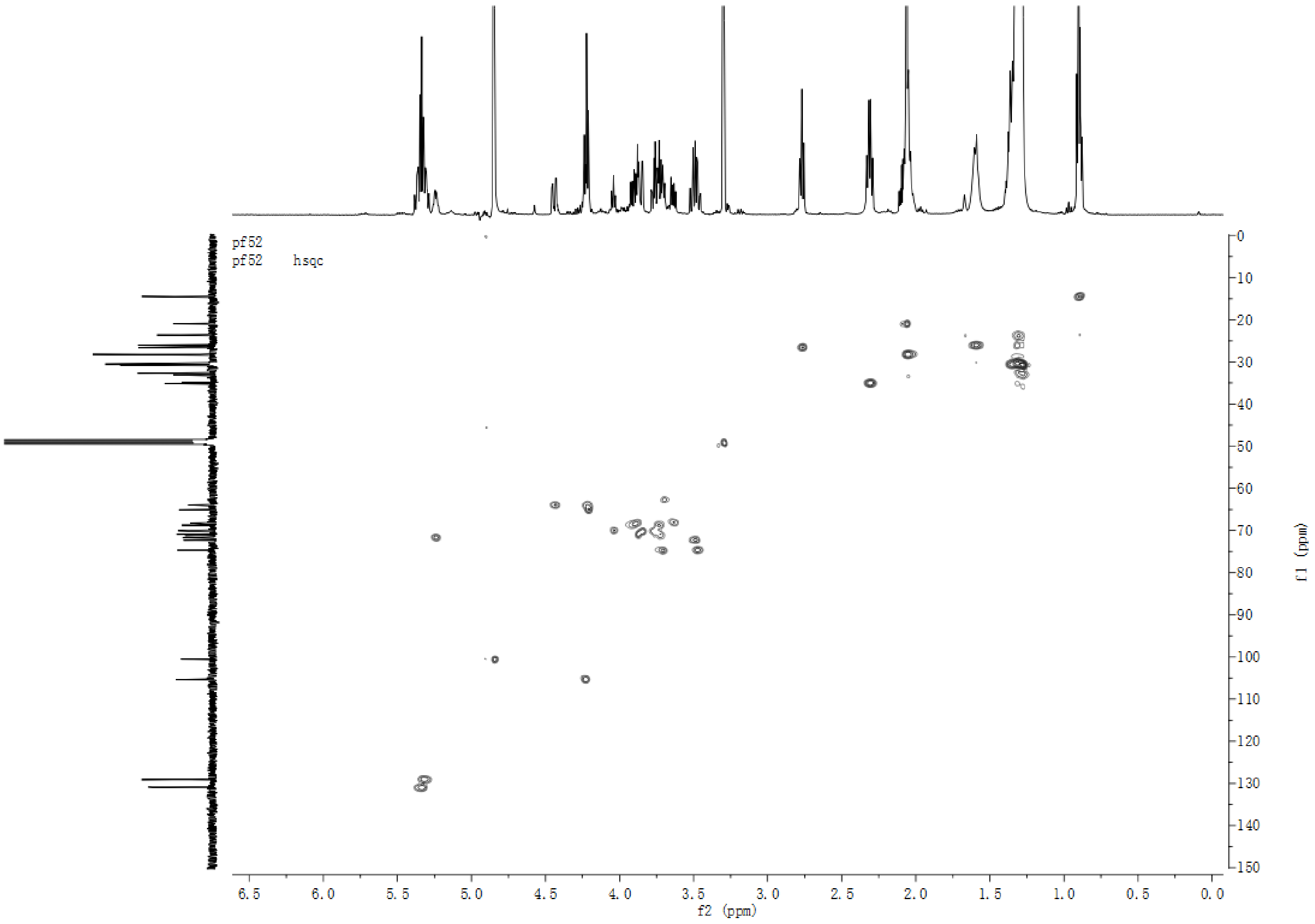

Figure S18 HSQC spectrum of compound **2**

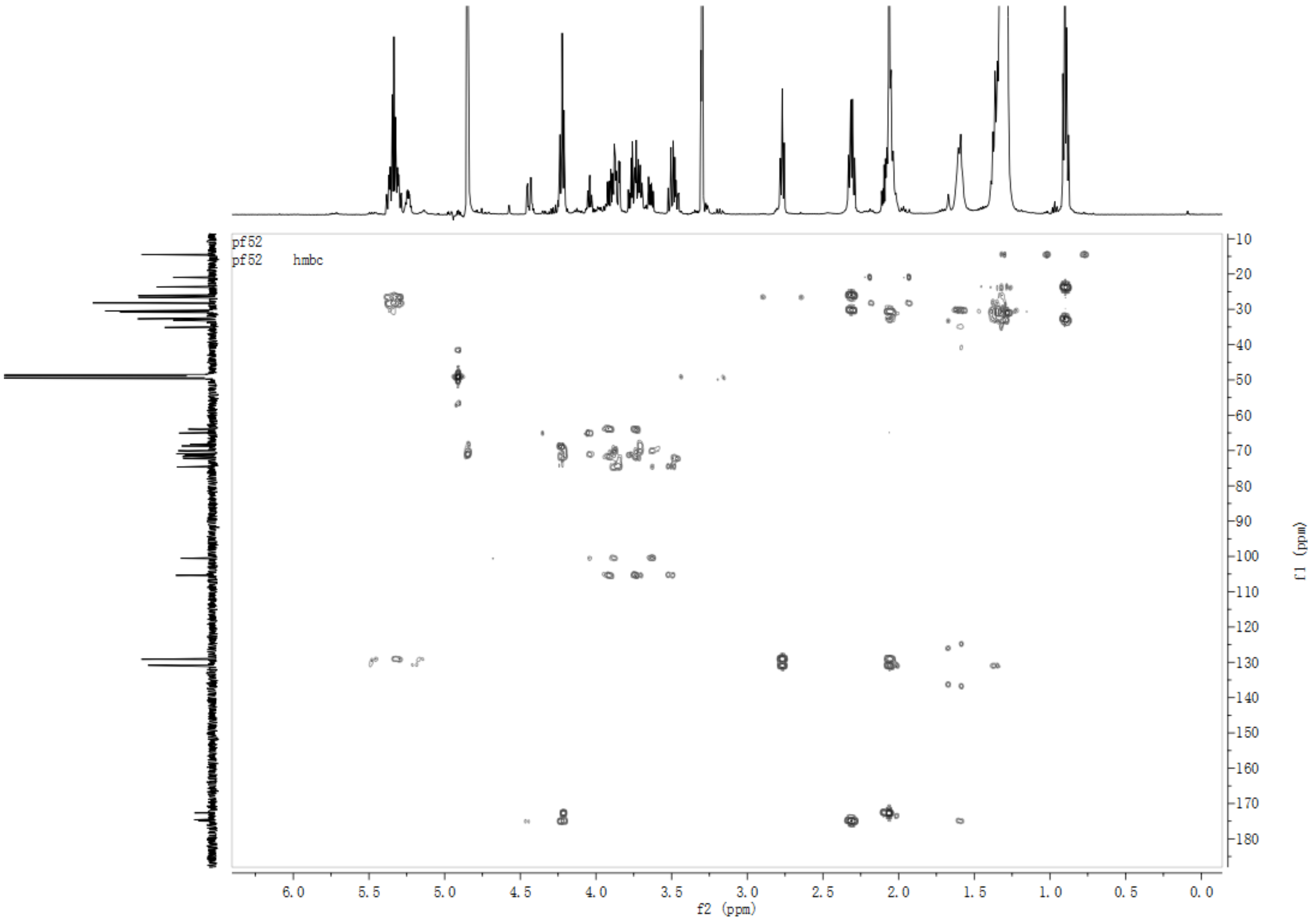

Figure S19 HMBC spectrum of compound **2**

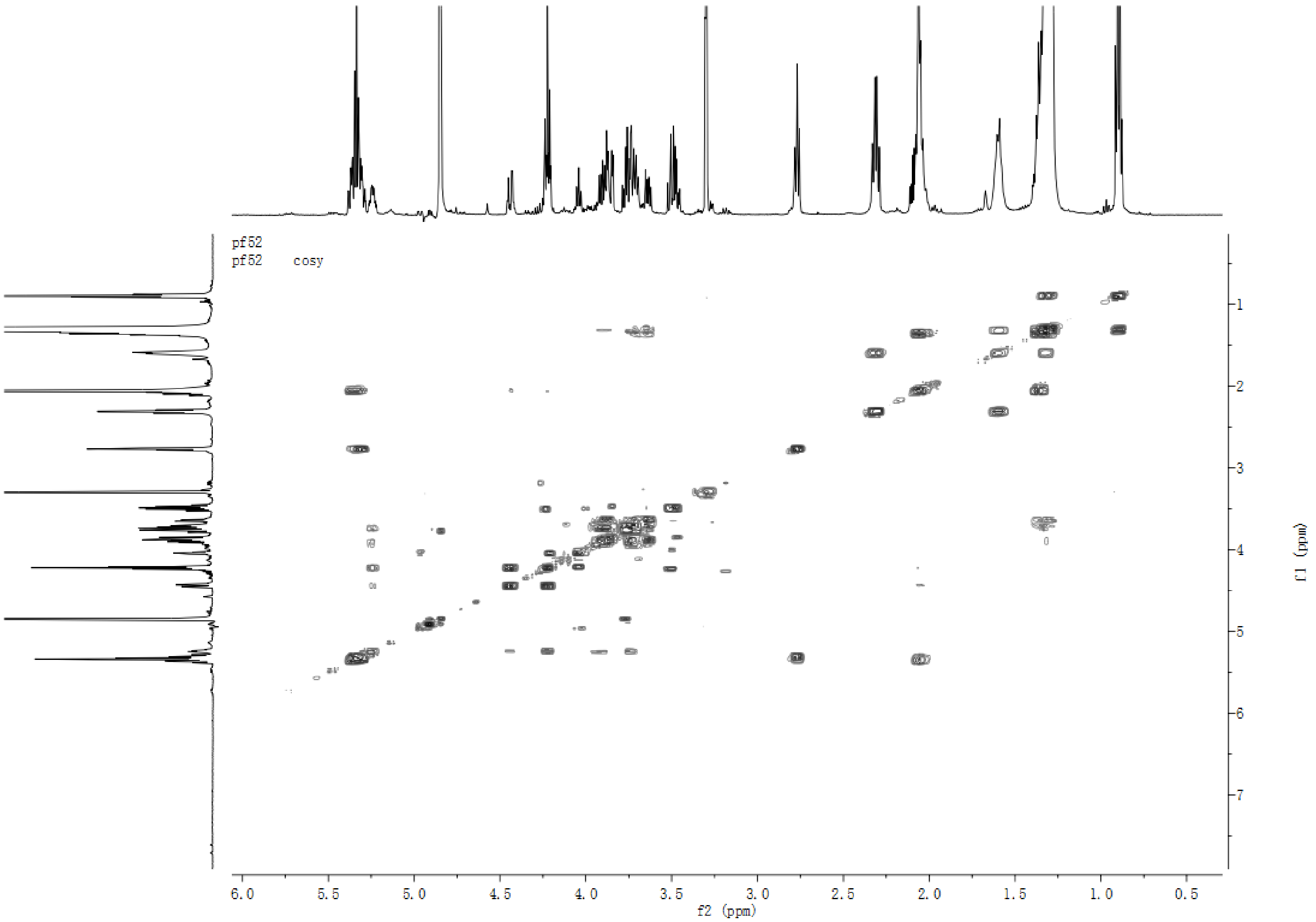

Figure S20 ^{1}H-^{1}H COSY spectrum of compound **2**

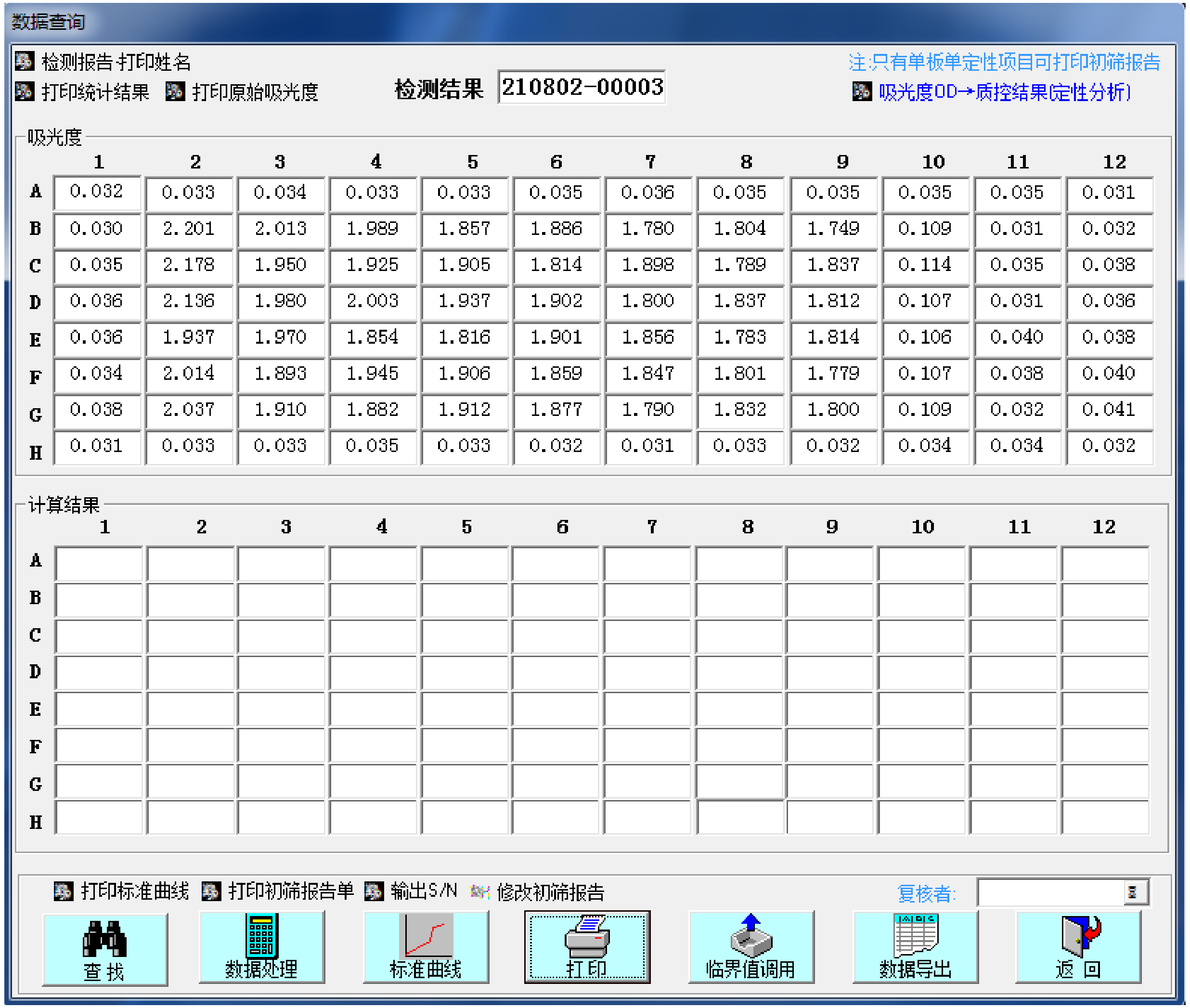

	1	2	3	4	5	6	7	8	9	10	11	12
A	0.032	0.033	0.034	0.033	0.033	0.035	0.036	0.035	0.035	0.035	0.035	0.031
B	0.030	2.201	2.013	1.989	1.857	1.886	1.780	1.804	1.749	0.109	0.031	0.032
C	0.035	2.178	1.950	1.925	1.905	1.814	1.898	1.789	1.837	0.114	0.035	0.038
D	0.036	2.136	1.980	2.003	1.937	1.902	1.800	1.837	1.812	0.107	0.031	0.036
E	0.036	1.937	1.970	1.854	1.816	1.901	1.856	1.783	1.814	0.106	0.040	0.038
F	0.034	2.014	1.893	1.945	1.906	1.859	1.847	1.801	1.779	0.107	0.038	0.040
G	0.038	2.037	1.910	1.882	1.912	1.877	1.790	1.832	1.800	0.109	0.032	0.041
H	0.031	0.033	0.033	0.035	0.033	0.032	0.031	0.033	0.032	0.034	0.034	0.032

	1	2	3	4	5	6	7	8	9	10	11	12
A												
B												
C												
D												
E												
F												
G												
H												

Figure S21 Cytotoxicity raw data of **1** and **2** against MCF-7/ADM

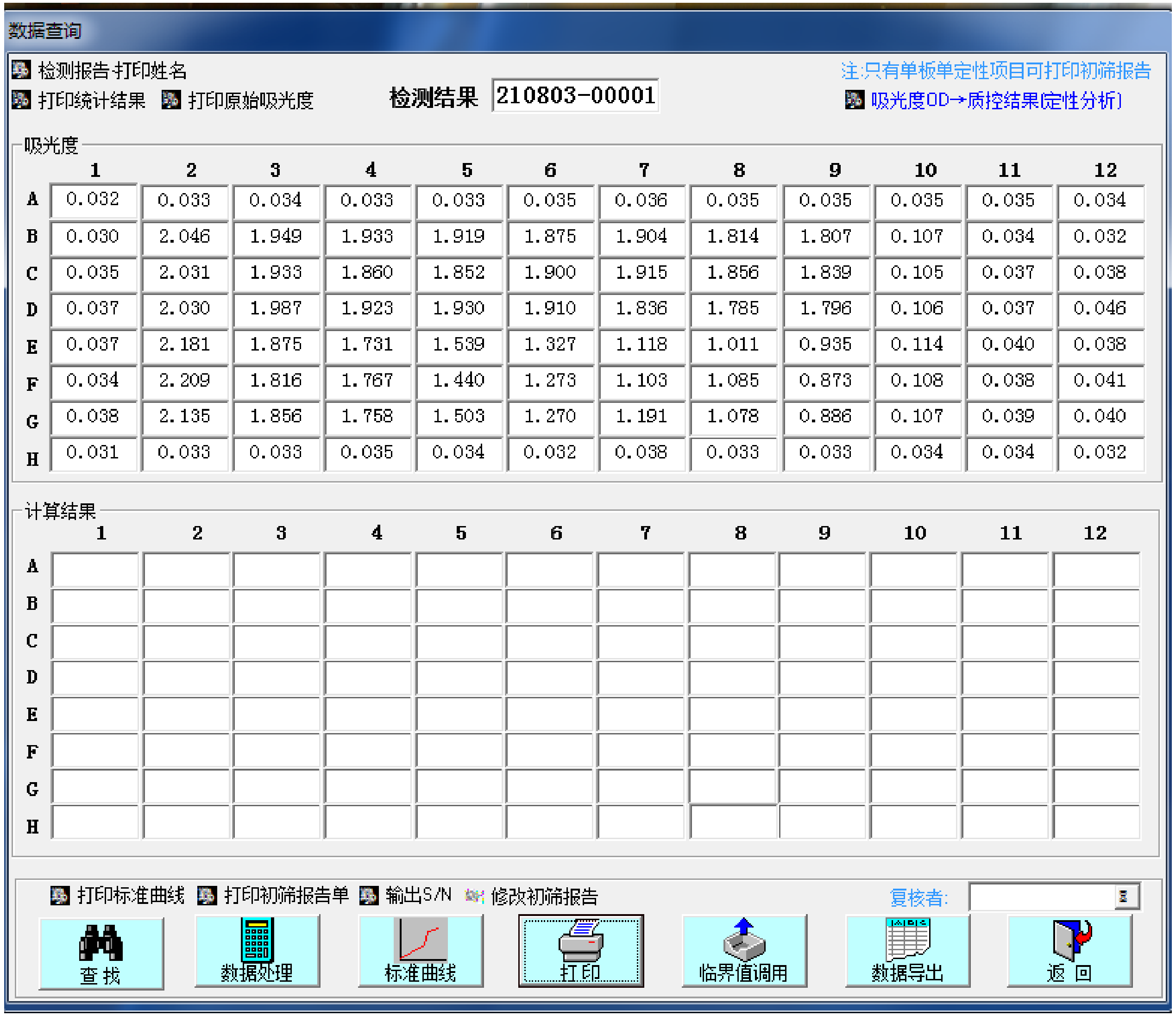

	1	2	3	4	5	6	7	8	9	10	11	12
A	0.032	0.033	0.034	0.033	0.033	0.035	0.036	0.035	0.035	0.035	0.035	0.034
B	0.030	2.046	1.949	1.933	1.919	1.875	1.904	1.814	1.807	0.107	0.034	0.032
C	0.035	2.031	1.933	1.860	1.852	1.900	1.915	1.856	1.839	0.105	0.037	0.038
D	0.037	2.030	1.987	1.923	1.930	1.910	1.836	1.785	1.796	0.106	0.037	0.046
E	0.037	2.181	1.875	1.731	1.539	1.327	1.118	1.011	0.935	0.114	0.040	0.038
F	0.034	2.209	1.816	1.767	1.440	1.273	1.103	1.085	0.873	0.108	0.038	0.041
G	0.038	2.135	1.856	1.758	1.503	1.270	1.191	1.078	0.886	0.107	0.039	0.040
H	0.031	0.033	0.033	0.035	0.034	0.032	0.038	0.033	0.033	0.034	0.034	0.032

Figure S22 Cytotoxicity raw data of ADM against MCF-7/ADM

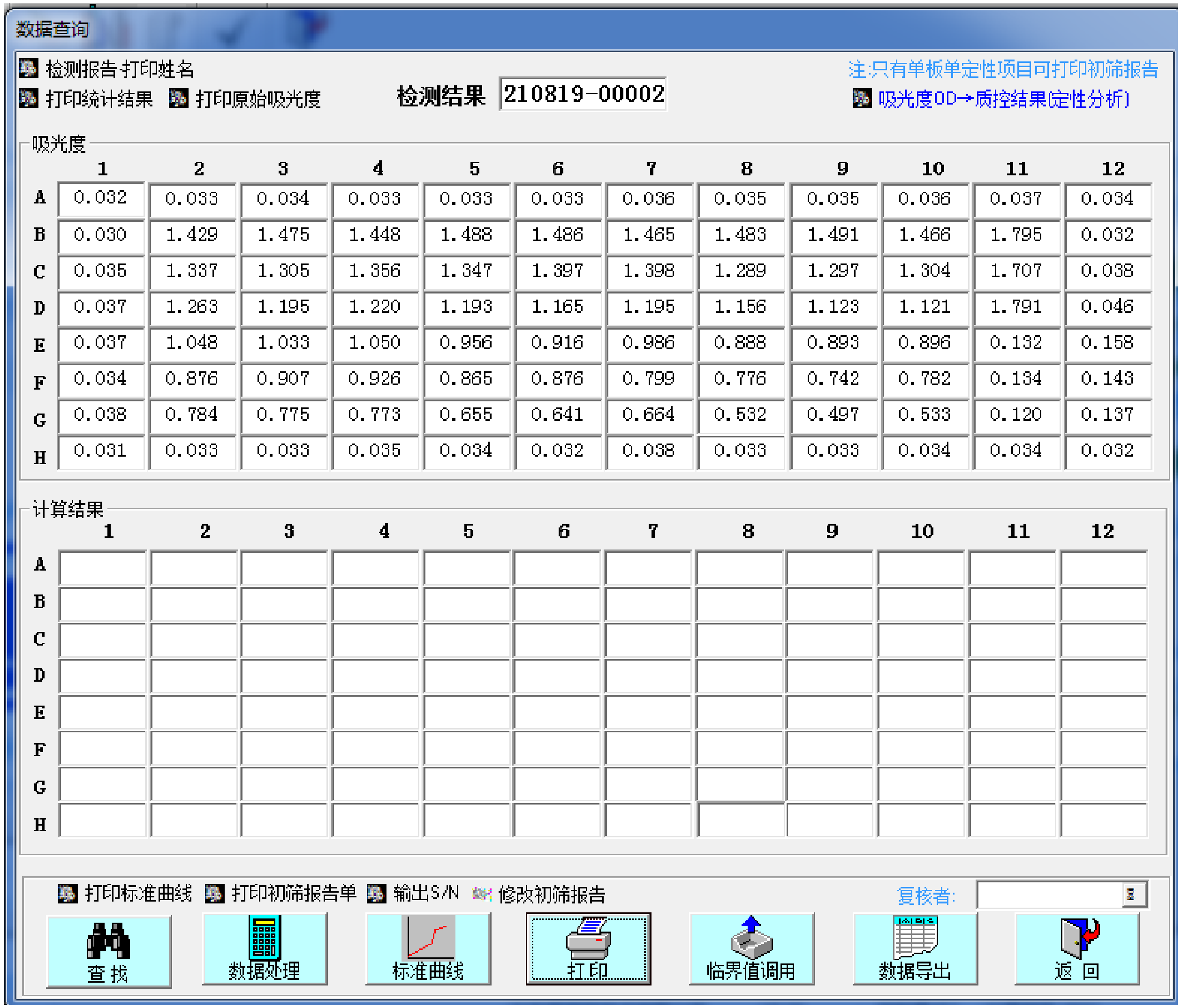

数据查询

检测报告打印姓名 注:只有单板单定性项目可打印初筛报告

打印统计结果 打印原始吸光度 检测结果 210819-00002 吸光度OD→质控结果(定性分析)

吸光度

	1	2	3	4	5	6	7	8	9	10	11	12
A	0.032	0.033	0.034	0.033	0.033	0.033	0.036	0.035	0.035	0.036	0.037	0.034
B	0.030	1.429	1.475	1.448	1.488	1.486	1.465	1.483	1.491	1.466	1.795	0.032
C	0.035	1.337	1.305	1.356	1.347	1.397	1.398	1.289	1.297	1.304	1.707	0.038
D	0.037	1.263	1.195	1.220	1.193	1.165	1.195	1.156	1.123	1.121	1.791	0.046
E	0.037	1.048	1.033	1.050	0.956	0.916	0.986	0.888	0.893	0.896	0.132	0.158
F	0.034	0.876	0.907	0.926	0.865	0.876	0.799	0.776	0.742	0.782	0.134	0.143
G	0.038	0.784	0.775	0.773	0.655	0.641	0.664	0.532	0.497	0.533	0.120	0.137
H	0.031	0.033	0.033	0.035	0.034	0.032	0.038	0.033	0.033	0.034	0.034	0.032

计算结果

	1	2	3	4	5	6	7	8	9	10	11	12
A												
B												
C												
D												
E												
F												
G												
H												

打印标准曲线 打印初筛报告单 输出S/N 修改初筛报告 复核者:

查找 数据处理 标准曲线 打印 临界值调用 数据导出 返回

Figure S23 Reversed MDR activity raw data of **1** against MCF-7/ADM

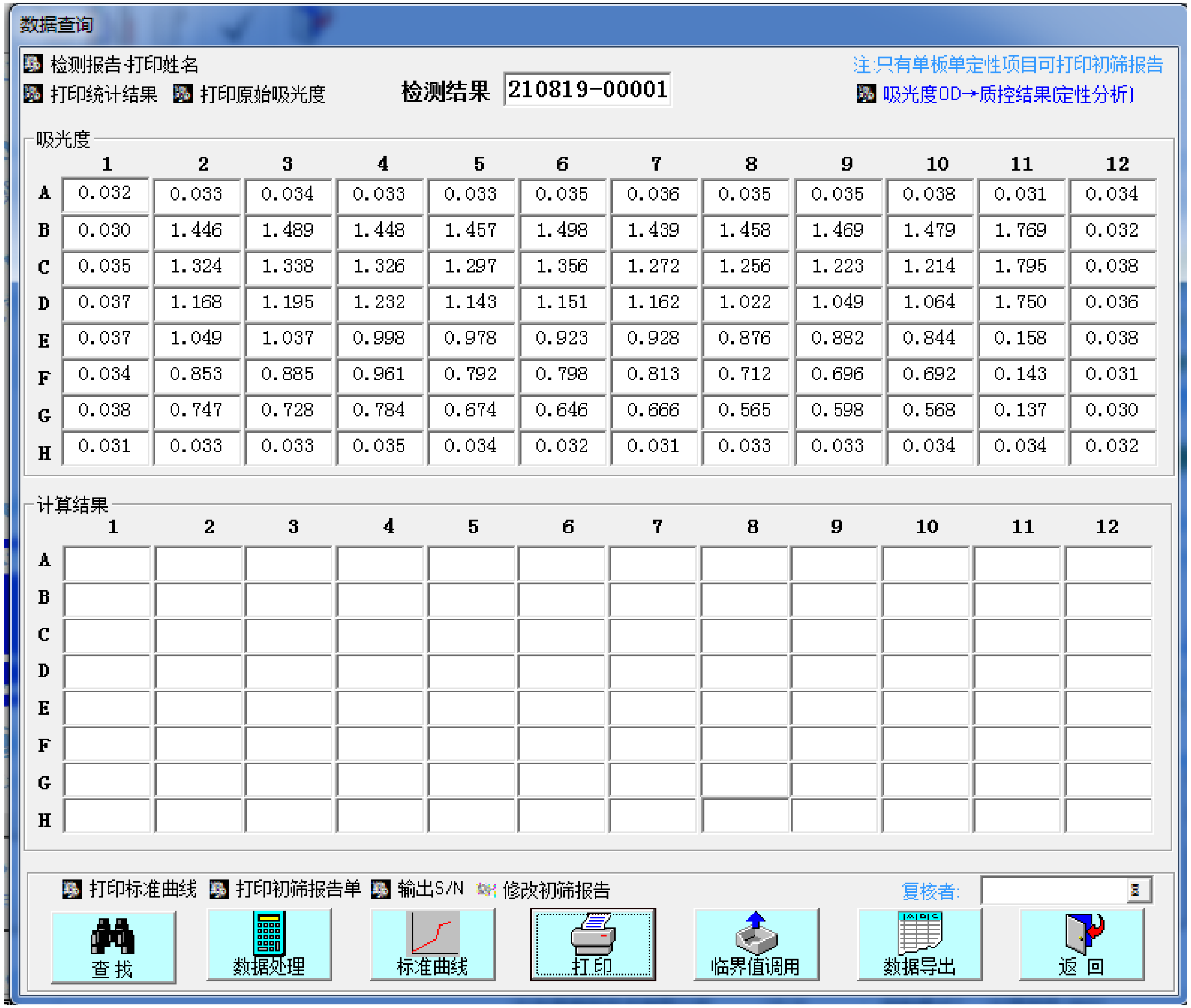

吸光度

	1	2	3	4	5	6	7	8	9	10	11	12
A	0.032	0.033	0.034	0.033	0.033	0.035	0.036	0.035	0.035	0.038	0.031	0.034
B	0.030	1.446	1.489	1.448	1.457	1.498	1.439	1.458	1.469	1.479	1.769	0.032
C	0.035	1.324	1.338	1.326	1.297	1.356	1.272	1.256	1.223	1.214	1.795	0.038
D	0.037	1.168	1.195	1.232	1.143	1.151	1.162	1.022	1.049	1.064	1.750	0.036
E	0.037	1.049	1.037	0.998	0.978	0.923	0.928	0.876	0.882	0.844	0.158	0.038
F	0.034	0.853	0.885	0.961	0.792	0.798	0.813	0.712	0.696	0.692	0.143	0.031
G	0.038	0.747	0.728	0.784	0.674	0.646	0.666	0.565	0.598	0.568	0.137	0.030
H	0.031	0.033	0.033	0.035	0.034	0.032	0.031	0.033	0.033	0.034	0.034	0.032

计算结果

	1	2	3	4	5	6	7	8	9	10	11	12
A												
B												
C												
D												
E												
F												
G												
H												

Figure S24 Reversed MDR activity raw data of **2** against MCF-7/ADM

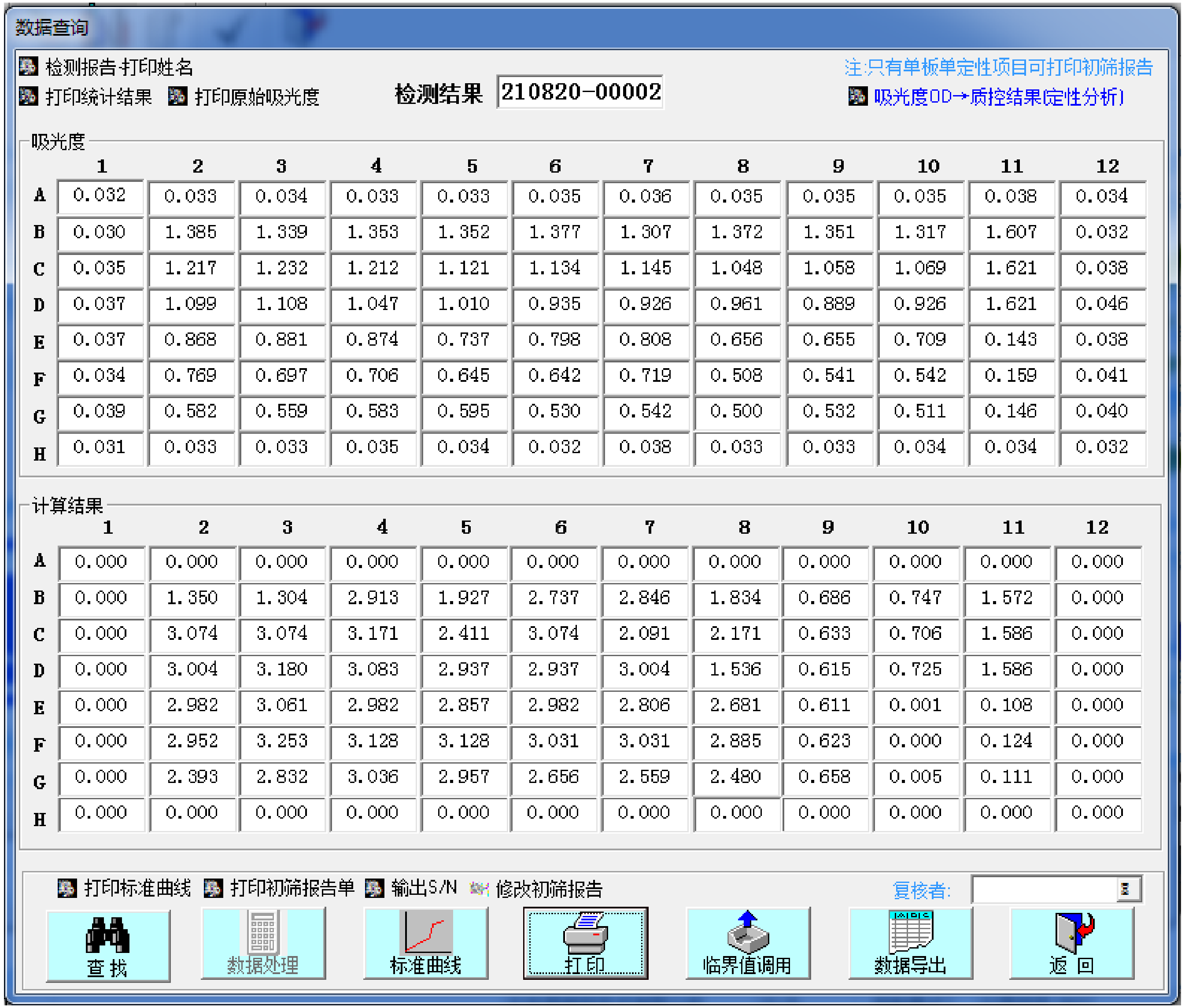

	1	2	3	4	5	6	7	8	9	10	11	12
A	0.032	0.033	0.034	0.033	0.033	0.035	0.036	0.035	0.035	0.035	0.038	0.034
B	0.030	1.385	1.339	1.353	1.352	1.377	1.307	1.372	1.351	1.317	1.607	0.032
C	0.035	1.217	1.232	1.212	1.121	1.134	1.145	1.048	1.058	1.069	1.621	0.038
D	0.037	1.099	1.108	1.047	1.010	0.935	0.926	0.961	0.889	0.926	1.621	0.046
E	0.037	0.868	0.881	0.874	0.737	0.798	0.808	0.656	0.655	0.709	0.143	0.038
F	0.034	0.769	0.697	0.706	0.645	0.642	0.719	0.508	0.541	0.542	0.159	0.041
G	0.039	0.582	0.559	0.583	0.595	0.530	0.542	0.500	0.532	0.511	0.146	0.040
H	0.031	0.033	0.033	0.035	0.034	0.032	0.038	0.033	0.033	0.034	0.034	0.032

计算结果

	1	2	3	4	5	6	7	8	9	10	11	12
A	0.000	0.000	0.000	0.000	0.000	0.000	0.000	0.000	0.000	0.000	0.000	0.000
B	0.000	1.350	1.304	2.913	1.927	2.737	2.846	1.834	0.686	0.747	1.572	0.000
C	0.000	3.074	3.074	3.171	2.411	3.074	2.091	2.171	0.633	0.706	1.586	0.000
D	0.000	3.004	3.180	3.083	2.937	2.937	3.004	1.536	0.615	0.725	1.586	0.000
E	0.000	2.982	3.061	2.982	2.857	2.982	2.806	2.681	0.611	0.001	0.108	0.000
F	0.000	2.952	3.253	3.128	3.128	3.031	3.031	2.885	0.623	0.000	0.124	0.000
G	0.000	2.393	2.832	3.036	2.957	2.656	2.559	2.480	0.658	0.005	0.111	0.000
H	0.000	0.000	0.000	0.000	0.000	0.000	0.000	0.000	0.000	0.000	0.000	0.000

Figure S25 Reversed MDR activity raw data of Vrp against MCF-7/ADM

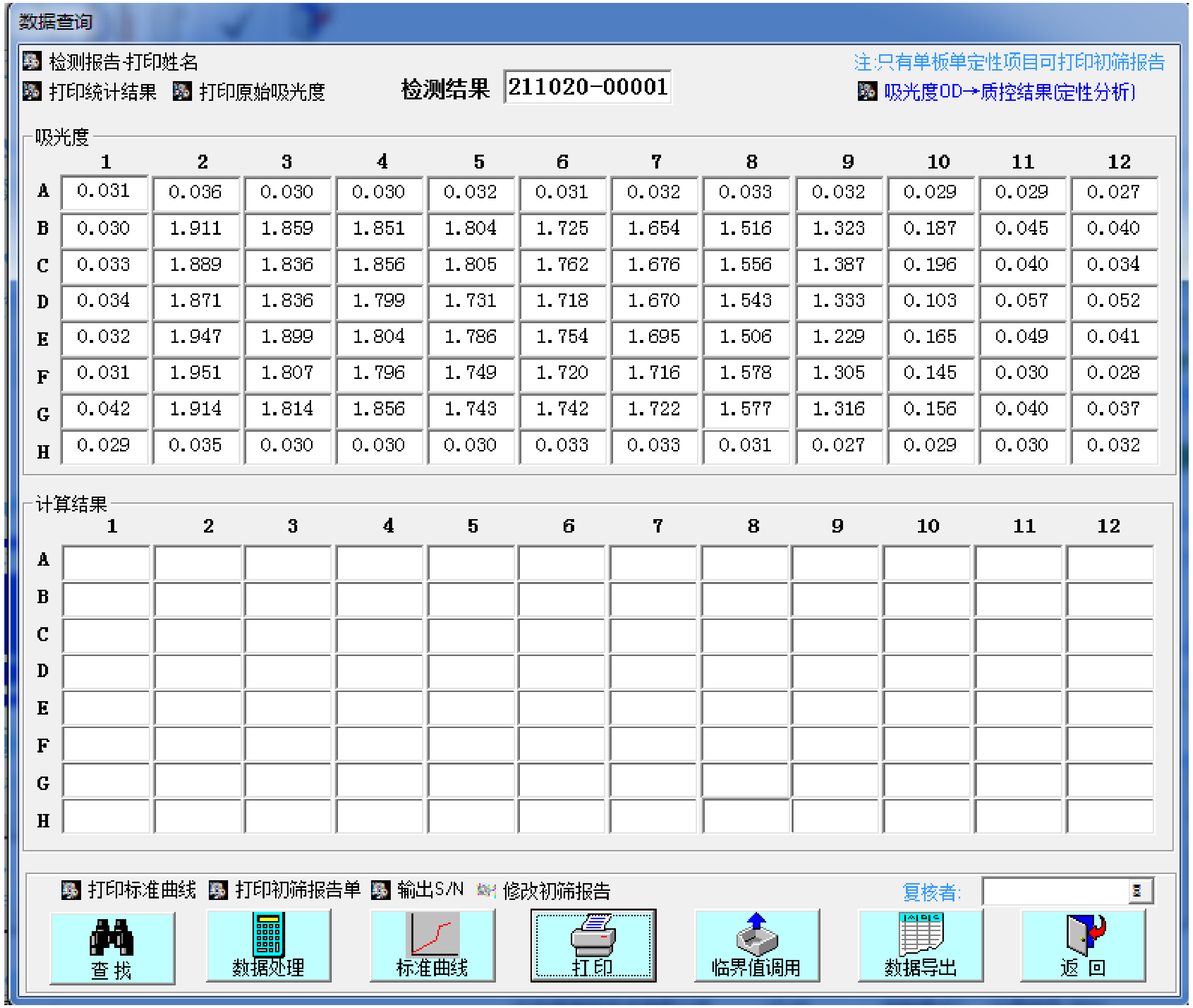

	1	2	3	4	5	6	7	8	9	10	11	12
A	0.031	0.036	0.030	0.030	0.032	0.031	0.032	0.033	0.032	0.029	0.029	0.027
B	0.030	1.911	1.859	1.851	1.804	1.725	1.654	1.516	1.323	0.187	0.045	0.040
C	0.033	1.889	1.836	1.856	1.805	1.762	1.676	1.556	1.387	0.196	0.040	0.034
D	0.034	1.871	1.836	1.799	1.731	1.718	1.670	1.543	1.333	0.103	0.057	0.052
E	0.032	1.947	1.899	1.804	1.786	1.754	1.695	1.506	1.229	0.165	0.049	0.041
F	0.031	1.951	1.807	1.796	1.749	1.720	1.716	1.578	1.305	0.145	0.030	0.028
G	0.042	1.914	1.814	1.856	1.743	1.742	1.722	1.577	1.316	0.156	0.040	0.037
H	0.029	0.035	0.030	0.030	0.030	0.033	0.033	0.031	0.027	0.029	0.030	0.032

Figure S26 Cytotoxicity raw data of **1** and **2** against A549/ADM

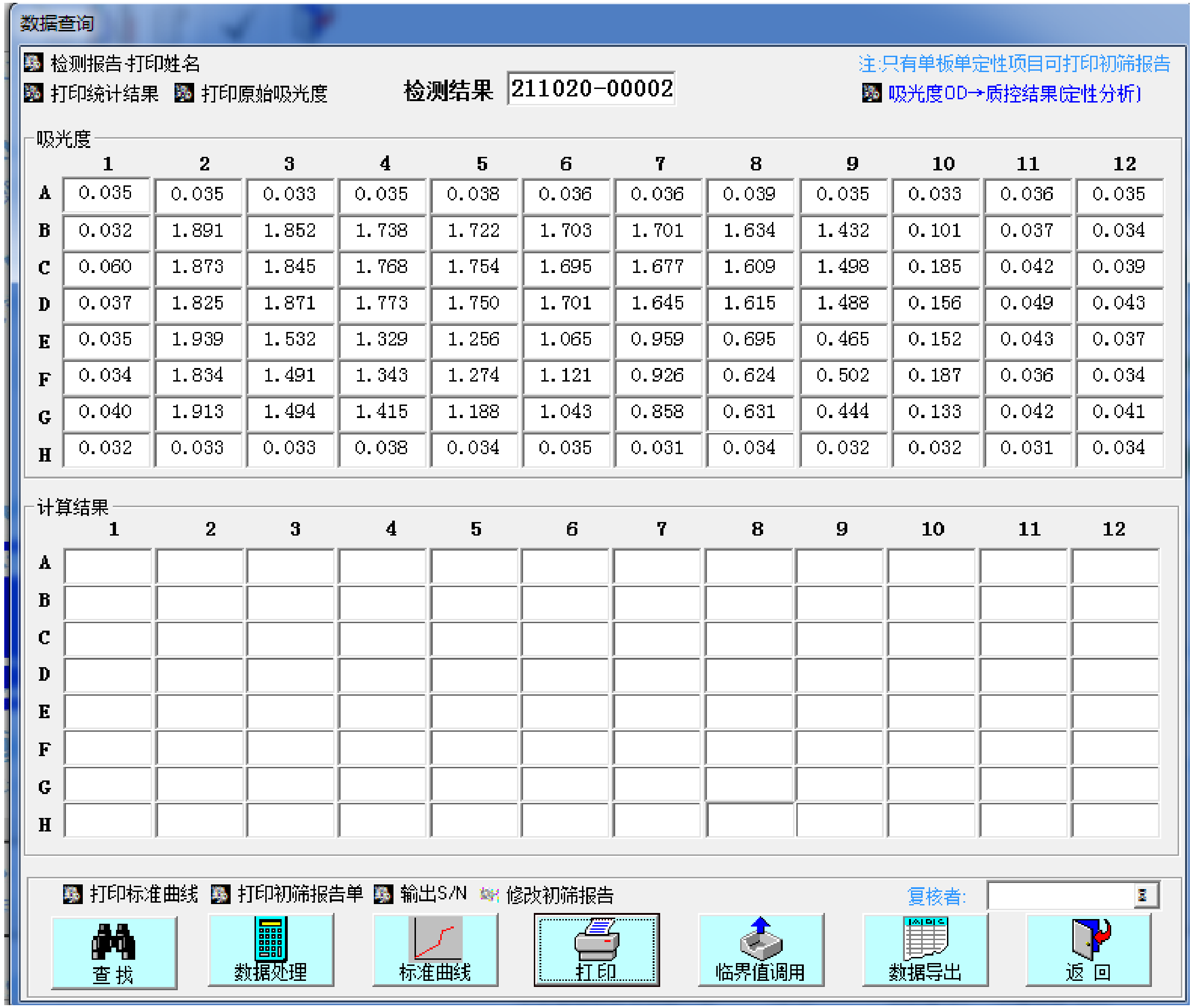

吸光度

	1	2	3	4	5	6	7	8	9	10	11	12
A	0.035	0.035	0.033	0.035	0.038	0.036	0.036	0.039	0.035	0.033	0.036	0.035
B	0.032	1.891	1.852	1.738	1.722	1.703	1.701	1.634	1.432	0.101	0.037	0.034
C	0.060	1.873	1.845	1.768	1.754	1.695	1.677	1.609	1.498	0.185	0.042	0.039
D	0.037	1.825	1.871	1.773	1.750	1.701	1.645	1.615	1.488	0.156	0.049	0.043
E	0.035	1.939	1.532	1.329	1.256	1.065	0.959	0.695	0.465	0.152	0.043	0.037
F	0.034	1.834	1.491	1.343	1.274	1.121	0.926	0.624	0.502	0.187	0.036	0.034
G	0.040	1.913	1.494	1.415	1.188	1.043	0.858	0.631	0.444	0.133	0.042	0.041
H	0.032	0.033	0.033	0.038	0.034	0.035	0.031	0.034	0.032	0.032	0.031	0.034

计算结果

	1	2	3	4	5	6	7	8	9	10	11	12
A												
B												
C												
D												
E												
F												
G												
H												

Figure S27 Cytotoxicity raw data of ADM against A549/ADM

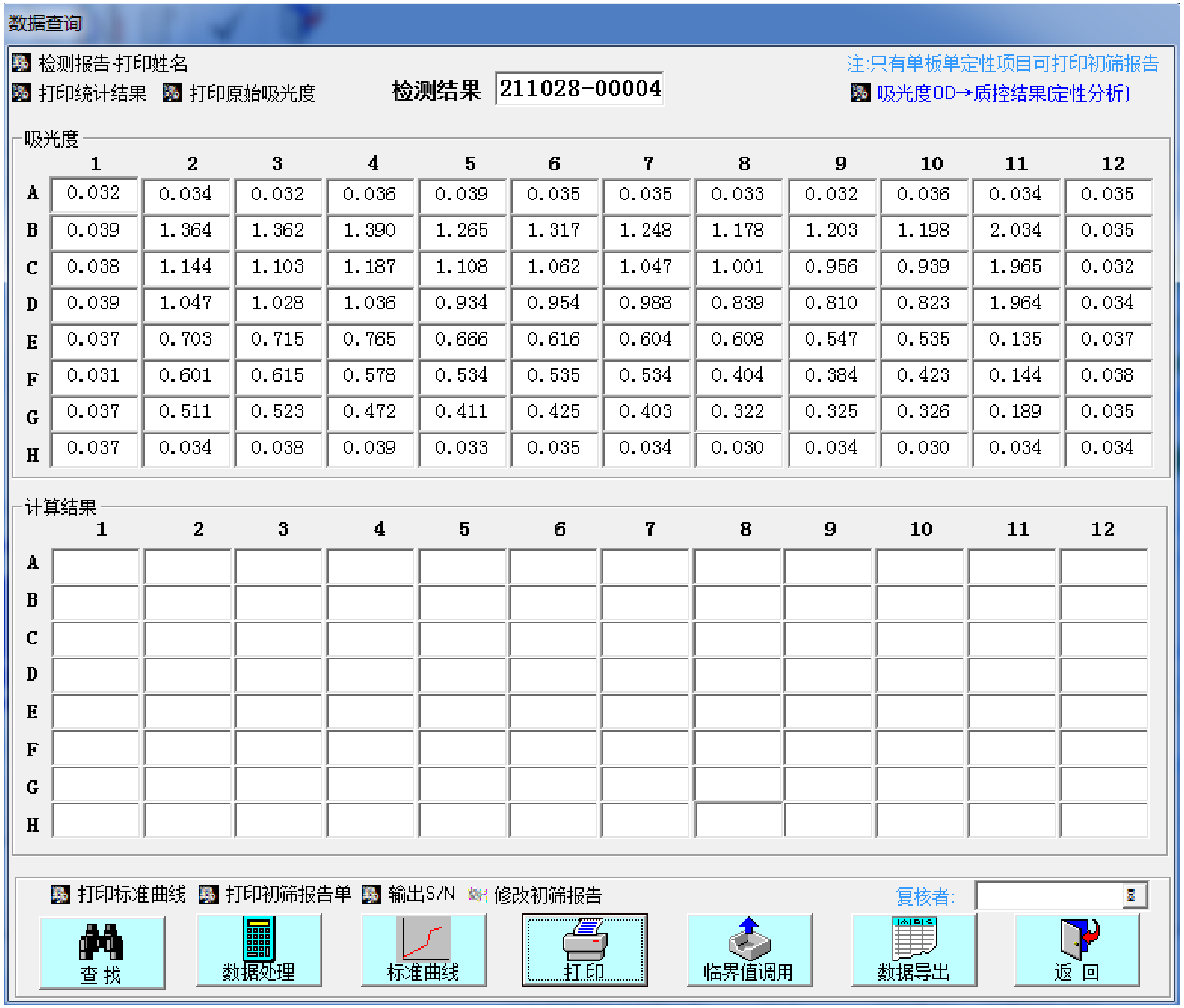

	1	2	3	4	5	6	7	8	9	10	11	12
A	0.032	0.034	0.032	0.036	0.039	0.035	0.035	0.033	0.032	0.036	0.034	0.035
B	0.039	1.364	1.362	1.390	1.265	1.317	1.248	1.178	1.203	1.198	2.034	0.035
C	0.038	1.144	1.103	1.187	1.108	1.062	1.047	1.001	0.956	0.939	1.965	0.032
D	0.039	1.047	1.028	1.036	0.934	0.954	0.988	0.839	0.810	0.823	1.964	0.034
E	0.037	0.703	0.715	0.765	0.666	0.616	0.604	0.608	0.547	0.535	0.135	0.037
F	0.031	0.601	0.615	0.578	0.534	0.535	0.534	0.404	0.384	0.423	0.144	0.038
G	0.037	0.511	0.523	0.472	0.411	0.425	0.403	0.322	0.325	0.326	0.189	0.035
H	0.037	0.034	0.038	0.039	0.033	0.035	0.034	0.030	0.034	0.030	0.034	0.034

Figure S28 Reversed MDR activity raw data of **1** against A549/ADM

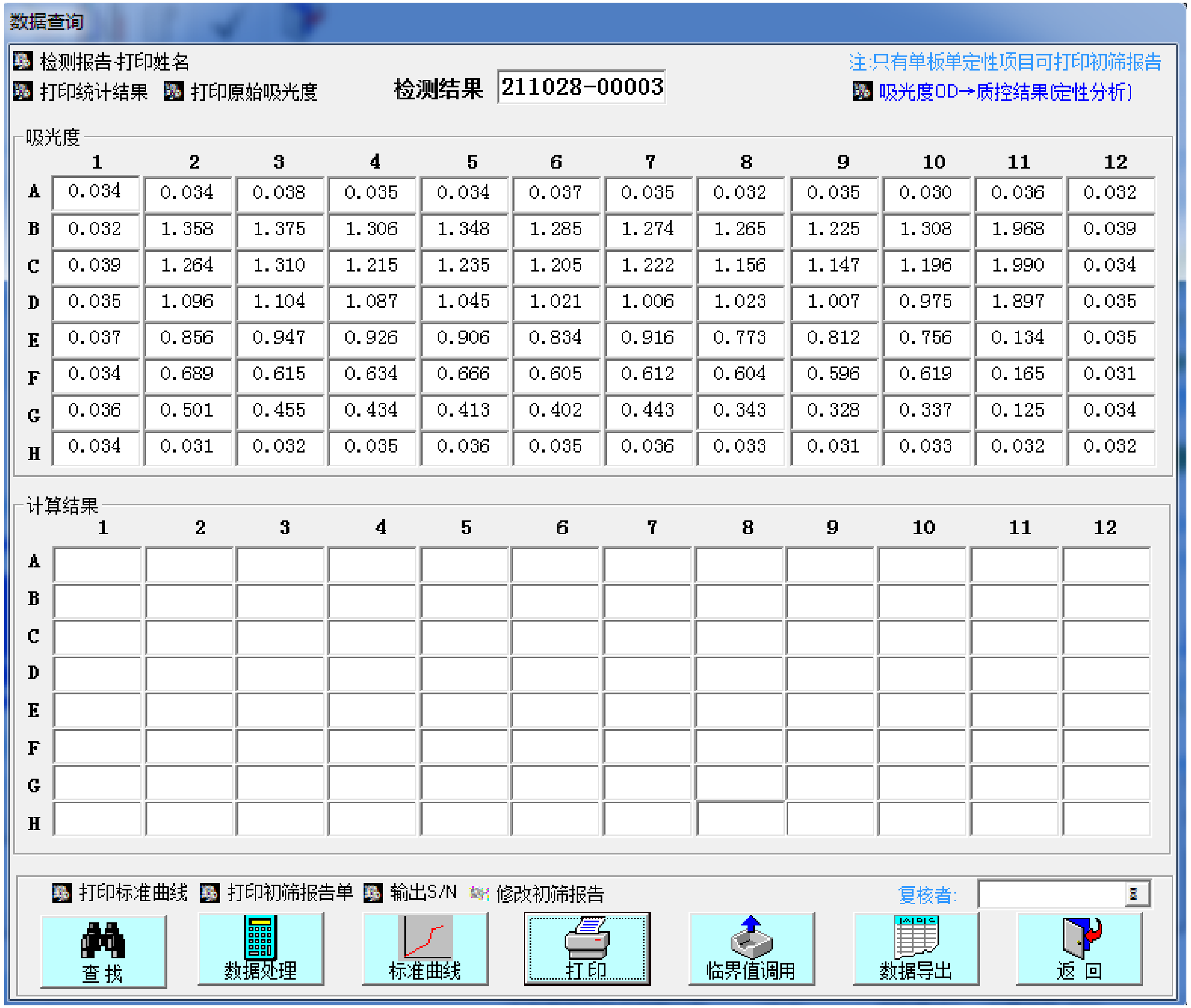

吸光度

	1	2	3	4	5	6	7	8	9	10	11	12
A	0.034	0.034	0.038	0.035	0.034	0.037	0.035	0.032	0.035	0.030	0.036	0.032
B	0.032	1.358	1.375	1.306	1.348	1.285	1.274	1.265	1.225	1.308	1.968	0.039
C	0.039	1.264	1.310	1.215	1.235	1.205	1.222	1.156	1.147	1.196	1.990	0.034
D	0.035	1.096	1.104	1.087	1.045	1.021	1.006	1.023	1.007	0.975	1.897	0.035
E	0.037	0.856	0.947	0.926	0.906	0.834	0.916	0.773	0.812	0.756	0.134	0.035
F	0.034	0.689	0.615	0.634	0.666	0.605	0.612	0.604	0.596	0.619	0.165	0.031
G	0.036	0.501	0.455	0.434	0.413	0.402	0.443	0.343	0.328	0.337	0.125	0.034
H	0.034	0.031	0.032	0.035	0.036	0.035	0.036	0.033	0.031	0.033	0.032	0.032

Figure S29 Reversed MDR activity raw data of **2** against A549/ADM

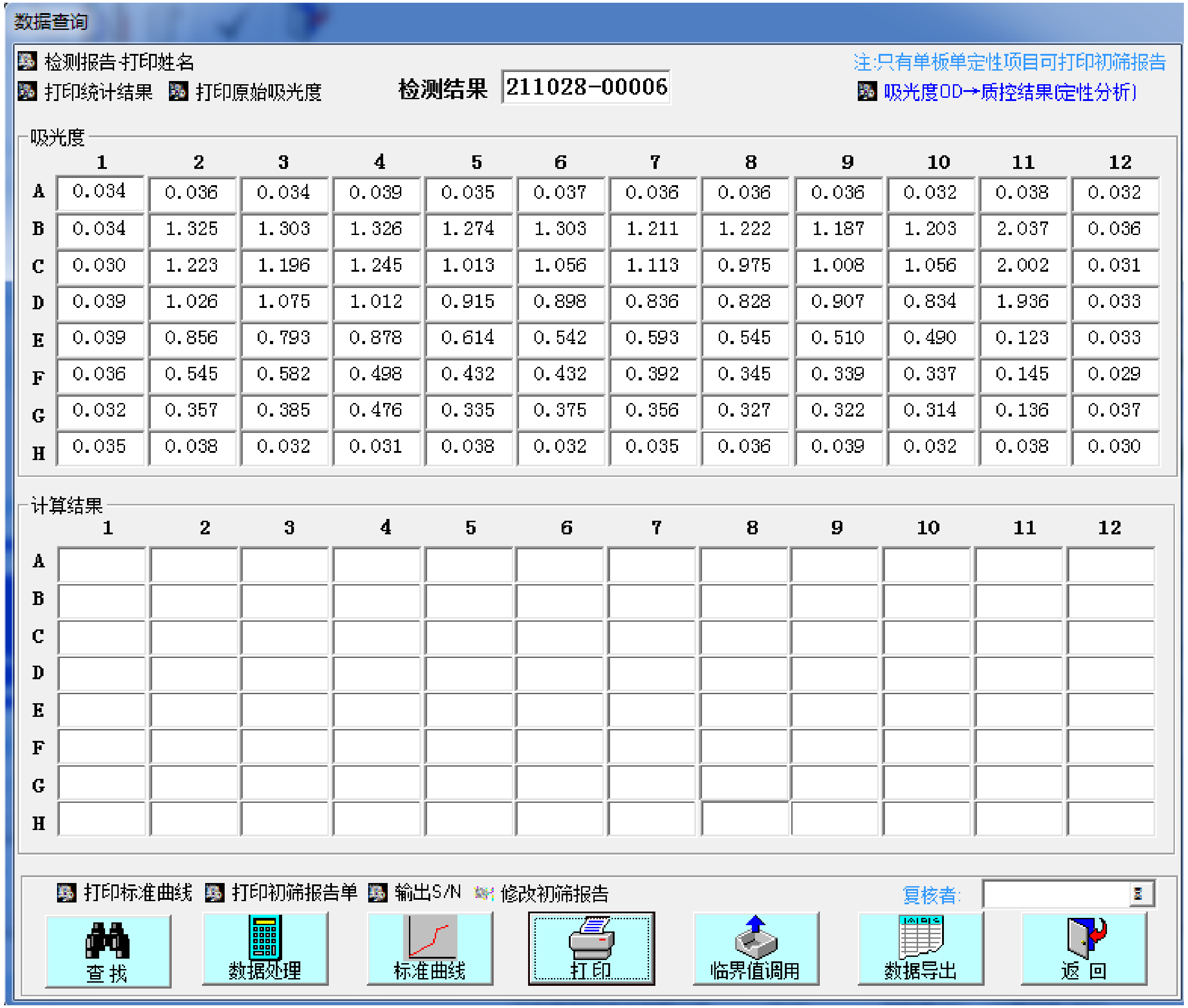

	1	2	3	4	5	6	7	8	9	10	11	12
A	0.034	0.036	0.034	0.039	0.035	0.037	0.036	0.036	0.036	0.032	0.038	0.032
B	0.034	1.325	1.303	1.326	1.274	1.303	1.211	1.222	1.187	1.203	2.037	0.036
C	0.030	1.223	1.196	1.245	1.013	1.056	1.113	0.975	1.008	1.056	2.002	0.031
D	0.039	1.026	1.075	1.012	0.915	0.898	0.836	0.828	0.907	0.834	1.936	0.033
E	0.039	0.856	0.793	0.878	0.614	0.542	0.593	0.545	0.510	0.490	0.123	0.033
F	0.036	0.545	0.582	0.498	0.432	0.432	0.392	0.345	0.339	0.337	0.145	0.029
G	0.032	0.357	0.385	0.476	0.335	0.375	0.356	0.327	0.322	0.314	0.136	0.037
H	0.035	0.038	0.032	0.031	0.038	0.032	0.035	0.036	0.039	0.032	0.038	0.030

	1	2	3	4	5	6	7	8	9	10	11	12
A												
B												
C												
D												
E												
F												
G												
H												

Figure S30 Reversed MDR activity raw data of Vrp against A549/ADM

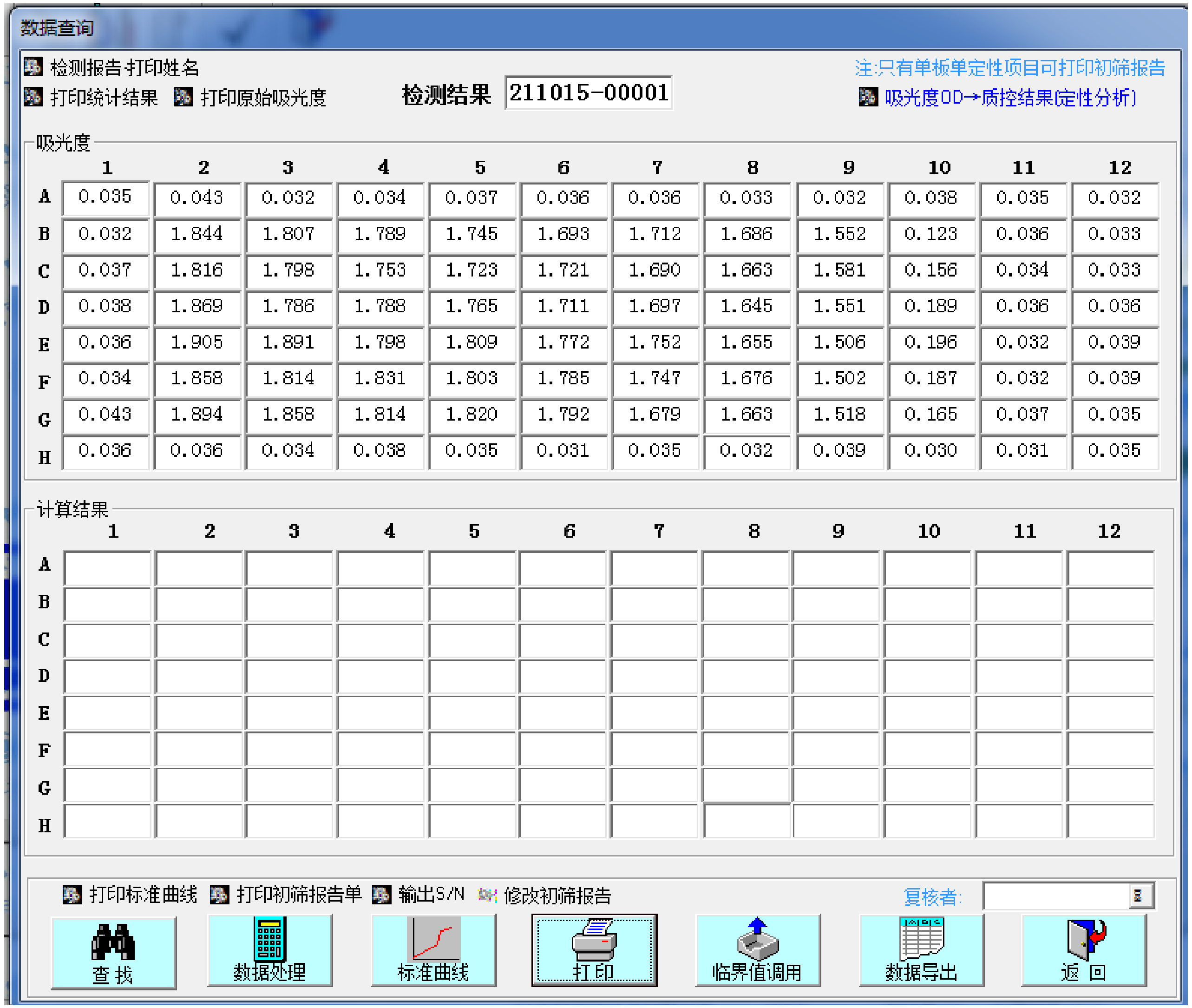

	1	2	3	4	5	6	7	8	9	10	11	12
A	0.035	0.043	0.032	0.034	0.037	0.036	0.036	0.033	0.032	0.038	0.035	0.032
B	0.032	1.844	1.807	1.789	1.745	1.693	1.712	1.686	1.552	0.123	0.036	0.033
C	0.037	1.816	1.798	1.753	1.723	1.721	1.690	1.663	1.581	0.156	0.034	0.033
D	0.038	1.869	1.786	1.788	1.765	1.711	1.697	1.645	1.551	0.189	0.036	0.036
E	0.036	1.905	1.891	1.798	1.809	1.772	1.752	1.655	1.506	0.196	0.032	0.039
F	0.034	1.858	1.814	1.831	1.803	1.785	1.747	1.676	1.502	0.187	0.032	0.039
G	0.043	1.894	1.858	1.814	1.820	1.792	1.679	1.663	1.518	0.165	0.037	0.035
H	0.036	0.036	0.034	0.038	0.035	0.031	0.035	0.032	0.039	0.030	0.031	0.035

Figure S31 Cytotoxicity raw data of **1** and **2** against K562/ADM

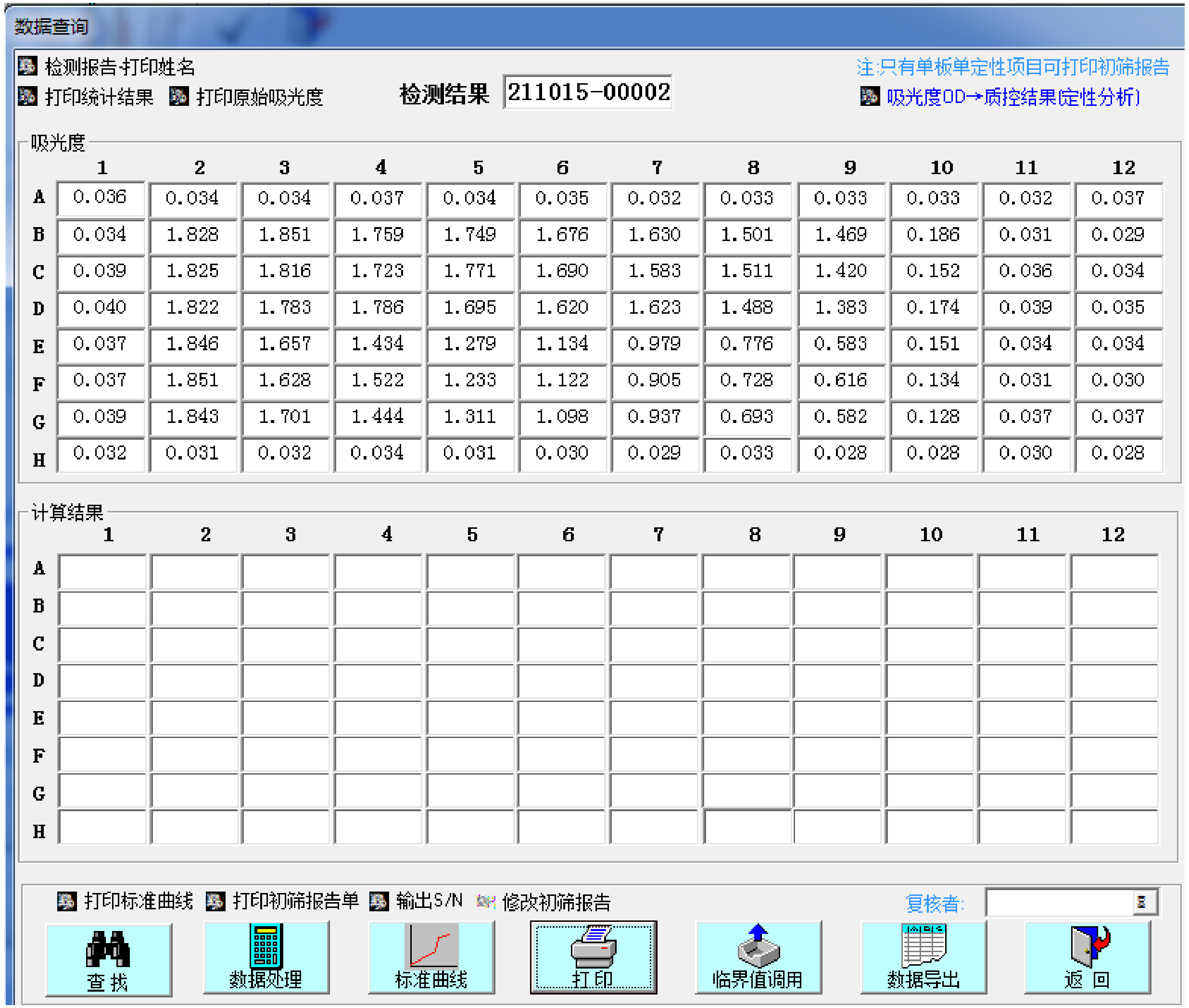

	1	2	3	4	5	6	7	8	9	10	11	12
A	0.036	0.034	0.034	0.037	0.034	0.035	0.032	0.033	0.033	0.033	0.032	0.037
B	0.034	1.828	1.851	1.759	1.749	1.676	1.630	1.501	1.469	0.186	0.031	0.029
C	0.039	1.825	1.816	1.723	1.771	1.690	1.583	1.511	1.420	0.152	0.036	0.034
D	0.040	1.822	1.783	1.786	1.695	1.620	1.623	1.488	1.383	0.174	0.039	0.035
E	0.037	1.846	1.657	1.434	1.279	1.134	0.979	0.776	0.583	0.151	0.034	0.034
F	0.037	1.851	1.628	1.522	1.233	1.122	0.905	0.728	0.616	0.134	0.031	0.030
G	0.039	1.843	1.701	1.444	1.311	1.098	0.937	0.693	0.582	0.128	0.037	0.037
H	0.032	0.031	0.032	0.034	0.031	0.030	0.029	0.033	0.028	0.028	0.030	0.028

Figure S32 Cytotoxicity raw data of ADM against K562/ADM

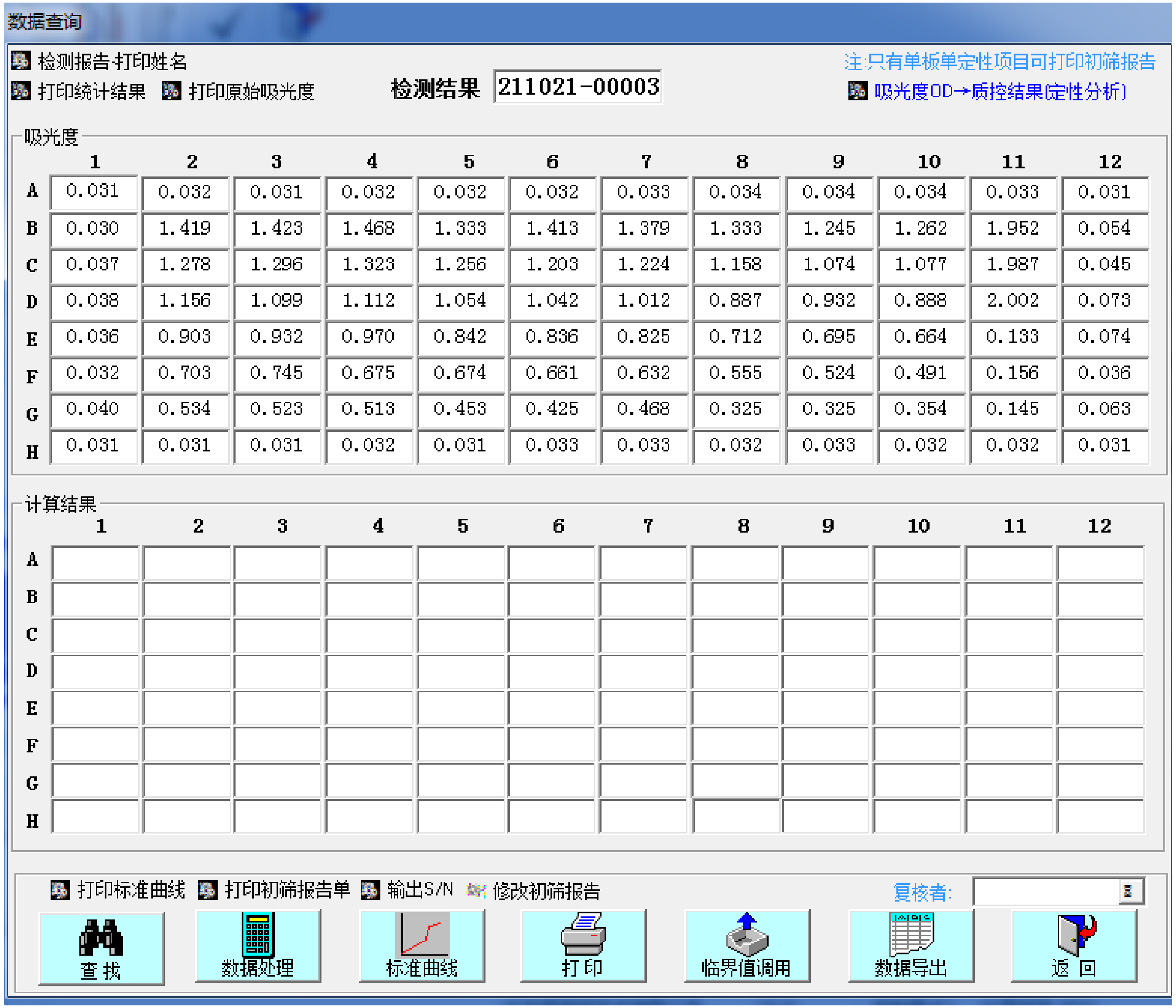
数据查询

检测报告打印姓名

打印统计结果 打印原始吸光度 检测结果 211021-00003

注:只有单板单定性项目可打印初筛报告

吸光度OD→质控结果(定性分析)

吸光度

	1	2	3	4	5	6	7	8	9	10	11	12
A	0.031	0.032	0.031	0.032	0.032	0.032	0.033	0.034	0.034	0.034	0.033	0.031
B	0.030	1.419	1.423	1.468	1.333	1.413	1.379	1.333	1.245	1.262	1.952	0.054
C	0.037	1.278	1.296	1.323	1.256	1.203	1.224	1.158	1.074	1.077	1.987	0.045
D	0.038	1.156	1.099	1.112	1.054	1.042	1.012	0.887	0.932	0.888	2.002	0.073
E	0.036	0.903	0.932	0.970	0.842	0.836	0.825	0.712	0.695	0.664	0.133	0.074
F	0.032	0.703	0.745	0.675	0.674	0.661	0.632	0.555	0.524	0.491	0.156	0.036
G	0.040	0.534	0.523	0.513	0.453	0.425	0.468	0.325	0.325	0.354	0.145	0.063
H	0.031	0.031	0.031	0.032	0.031	0.033	0.033	0.032	0.033	0.032	0.032	0.031

计算结果

	1	2	3	4	5	6	7	8	9	10	11	12
A												
B												
C												
D												
E												
F												
G												
H												

Figure S33 Reversed MDR activity raw data of **1** against K562/ADM

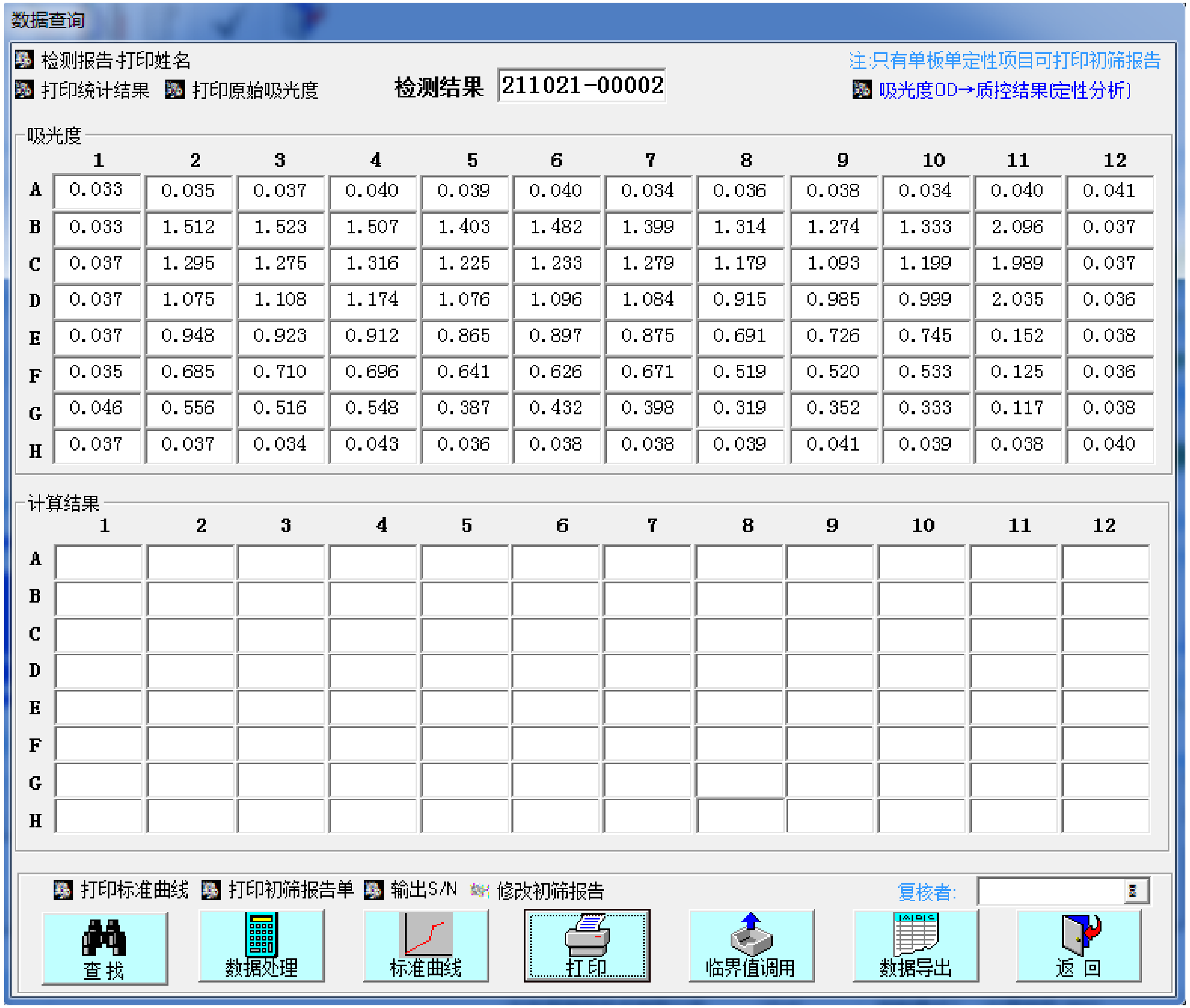

数据查询

检测报告 打印姓名

打印统计结果 打印原始吸光度 **检测结果** 211021-00002

注:只有单板单定性项目可打印初筛报告

吸光度OD→质控结果(定性分析)

吸光度

	1	2	3	4	5	6	7	8	9	10	11	12
A	0.033	0.035	0.037	0.040	0.039	0.040	0.034	0.036	0.038	0.034	0.040	0.041
B	0.033	1.512	1.523	1.507	1.403	1.482	1.399	1.314	1.274	1.333	2.096	0.037
C	0.037	1.295	1.275	1.316	1.225	1.233	1.279	1.179	1.093	1.199	1.989	0.037
D	0.037	1.075	1.108	1.174	1.076	1.096	1.084	0.915	0.985	0.999	2.035	0.036
E	0.037	0.948	0.923	0.912	0.865	0.897	0.875	0.691	0.726	0.745	0.152	0.038
F	0.035	0.685	0.710	0.696	0.641	0.626	0.671	0.519	0.520	0.533	0.125	0.036
G	0.046	0.556	0.516	0.548	0.387	0.432	0.398	0.319	0.352	0.333	0.117	0.038
H	0.037	0.037	0.034	0.043	0.036	0.038	0.038	0.039	0.041	0.039	0.038	0.040

计算结果

	1	2	3	4	5	6	7	8	9	10	11	12
A												
B												
C												
D												
E												
F												
G												
H												

打印标准曲线 打印初筛报告单 输出S/N 修改初筛报告 复核者:

查 找 数据处理 标准曲线 打 印 临界值调用 数据导出 返 回

Figure S34 Reversed MDR activity raw data of **2** against K562/ADM

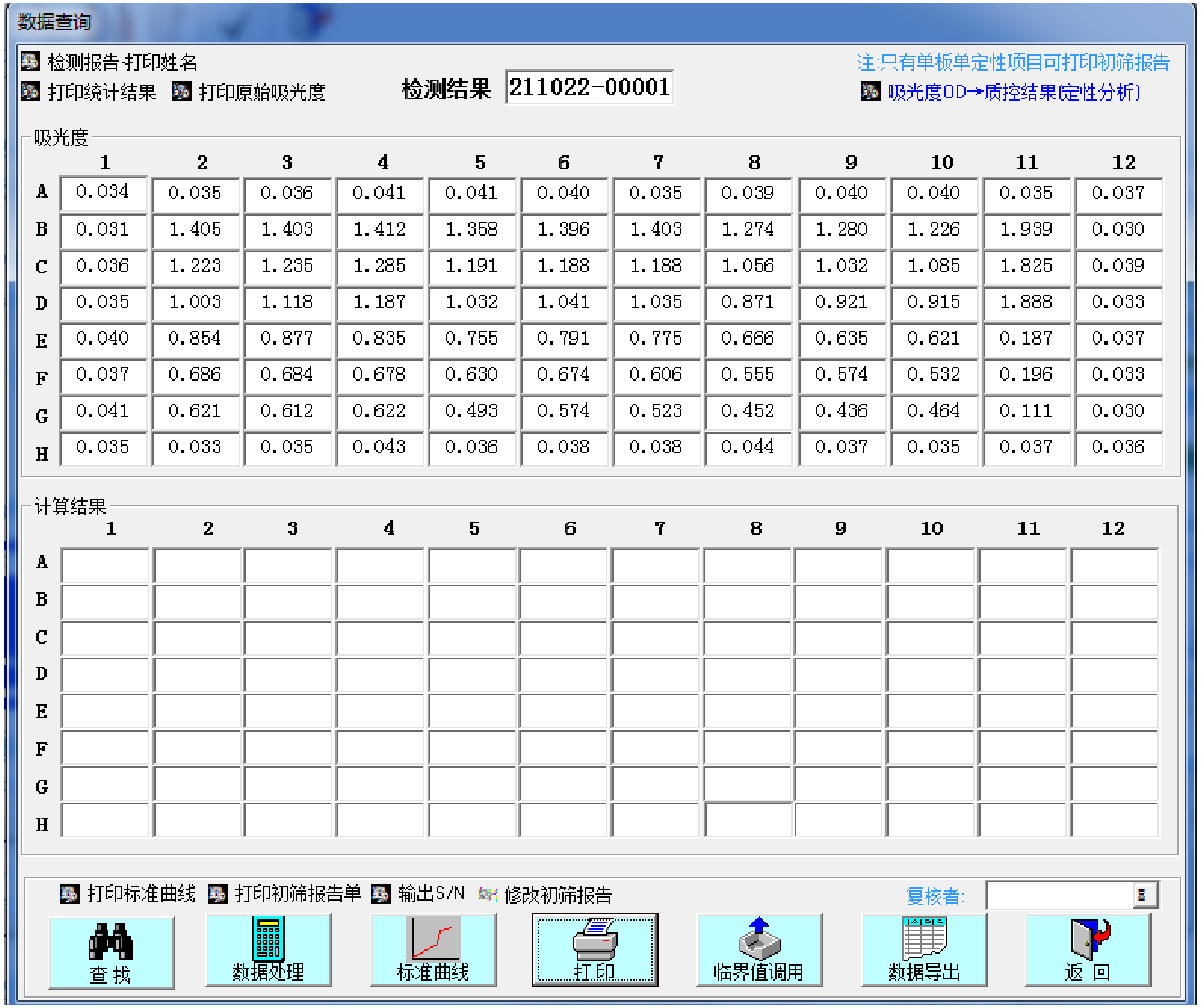

吸光度

	1	2	3	4	5	6	7	8	9	10	11	12
A	0.034	0.035	0.036	0.041	0.041	0.040	0.035	0.039	0.040	0.040	0.035	0.037
B	0.031	1.405	1.403	1.412	1.358	1.396	1.403	1.274	1.280	1.226	1.939	0.030
C	0.036	1.223	1.235	1.285	1.191	1.188	1.188	1.056	1.032	1.085	1.825	0.039
D	0.035	1.003	1.118	1.187	1.032	1.041	1.035	0.871	0.921	0.915	1.888	0.033
E	0.040	0.854	0.877	0.835	0.755	0.791	0.775	0.666	0.635	0.621	0.187	0.037
F	0.037	0.686	0.684	0.678	0.630	0.674	0.606	0.555	0.574	0.532	0.196	0.033
G	0.041	0.621	0.612	0.622	0.493	0.574	0.523	0.452	0.436	0.464	0.111	0.030
H	0.035	0.033	0.035	0.043	0.036	0.038	0.038	0.044	0.037	0.035	0.037	0.036

计算结果

	1	2	3	4	5	6	7	8	9	10	11	12
A												
B												
C												
D												
E												
F												
G												
H												

Figure S35 Reversed MDR activity raw data of Vrp against K562/ADM

Table S1. Spectroscopic data of compounds **3–18**

Compound	Reference	Description
1-*O*-palmitoyl-3-*O*-[*α*-*D*-galactopyranosyl-(1→6)-*β*-*D*-galactopyranosyl]- glycerol (**3**)	[1]	$C_{31}H_{58}O_{14}Na$(HR-ESI-MS *m/z* 677.37152 [*M* + Na]$^+$); ^{1}H NMR (500 MHz, Methanol-*d*$_4$) *δ*5.28-5.38 (4H, m, H-9‴, 10‴, 12‴, 13‴), 4.86 (1H, br s, H-1″), 4.24 (1H, d, *J* = 7.4 Hz, H-1′), 4.14 (2H, dd, *J* = 5.3, 3.3 Hz, H-3), 3.98 (1H, m, H-2), 3.82-3.91 (5H, m, H-1β, 4′, 2″, 4″, 6′β), 3.69-3.79 (5H, m, H-5′, 5″, 3″, 6″), 3.65 (2H, dd, *J* = 10.5, 4.6 Hz, H-1α, 6α), 3.54 (1H, m, H-2′), 3.50 (1H, m, H-3′), 2.77 (2H, t, *J* = 6.5 Hz, H-11‴), 2.35 (2H, t, *J* = 7.5 Hz, H-2‴), 2.06 (4H, m, H-8‴, 14‴), 1.61 (2H, m, H-3‴), 1.28-1.37 (14H, m, H-4‴, 5‴, 6‴, 7‴, 8‴, 15‴, 16‴, 17‴), 0.90 (3H, t, *J* = 7.0 Hz, H-18‴); ^{13}C NMR (126 MHz, Methanol-*d*$_4$) *δ*175.5 (s, C-1‴), 105.3 (d, C-1′), 100.5 (d, C-1″), 74.7 (d, C-3′), 74.6 (d, C-5′), 72.6 (d, C-2′), 72.5 (d, C-5″), 72.1 (t, C-1), 71.4 (d, C-3″), 71.0 (d, C-4″), 70.2 (d, C-2), 70.1 (d, C-4′), 69.7 (d, C-2″), 67.8 (t, C-6′), 66.6 (t, C-3), 62.7 (t, C-6″), 34.9 (t, C-2‴), 33.1 (t, C-16‴), 30.8-30.2 (t, C-15‴), 28.1 (t, C-8‴), 26.0 (t, C-3‴), 23.7 (t, C-17‴), 14.4 (q, C-18‴).
gingerglycolipid C (**4**)	[2]	$C_{33}H_{60}O_{14}Na$ (HR-ESI-MS *m/z* 703.38207 [*M* + Na]$^+$; ^{1}H NMR (500 MHz, Methanol-*d*$_4$) *δ*5.28-5.38 (2H, m, H-9‴, 10‴), 4.84 (1H, br s, H-1″), 4.24 (1H, d, *J* = 7.3 Hz, H-1′), 4.13 (2H, dd, *J* = 5.4, 3.1 Hz, H-3), 3.97 (1H, m, H-2), 3.81-3.90 (5H, m, H-1β, 4′, 2″, 4″, 6′β), 3.69-3.78 (5H, m, H-5′, 5″, 3″, 6″), 3.66 (2H, dd, *J* = 10.5, 4.5 Hz, H-1α, 6α), 3.52 (1H, dd, *J* = 10.0, 7.2 Hz, H-2′), 3.48 (1H, dd, *J* = 10.5, 6.8 Hz H-3′), 2.34 (2H, t, *J* = 7.5 Hz, H-2‴), 2.05 (4H, m, H-8‴, 11‴), 1.60 (2H, m, H-3‴), 1.27-1.36 (20H, m, H-4‴-7‴, 12‴-7‴), 0.89 (3H, t, *J*=6.8 Hz, H-18‴); ^{13}C NMR (126 MHz, Methanol-*d*$_4$) *δ*175.5 (s, C-1‴), 130.9 (d, C-9‴), 130.9 (d, C-10‴), 105.3 (d, C-1′), 100.5 (d, C-1″), 74.7 (d, C-3′), 74.6 (d, C-5′), 72.6 (d, C-2′), 72.5 (d, C-5″), 72.1 (t, C-1), 71.4 (d, C-3″), 71.0 (d, C-4″), 70.2 (d, C-2), 70.1 (d, C-4′), 69.7 (d, C-2″), 67.8 (t, C-6′), 66.6 (t, C-3), 62.7 (t, C-6″), 34.9 (t, C-2‴), 32.7 (t, C-16‴), 30.2-30.8 (t, C-4‴, 5‴,6‴, 7‴, 12‴, 13‴, 14‴, 15‴), 28.2 (t, C-8‴), 28.2 (t, C-11‴), 26.0 (t, C-3‴), 23.7 (t, C-17‴), 14.4 (q, C-18‴);
gingerglycolipid B (**5**)	[3]	$C_{33}H_{58}O_{14}Na$ (HR-ESI-MS *m/z* 701.36981 [*M* + Na]$^+$; ^{1}H NMR (500 MHz, Methanol-*d*$_4$) *δ*5.28-5.38 (4H, m, H-9‴, 10‴, 12‴, 13‴), 4.86 (1H, br s, H-1″), 4.24 (1H, d, *J* = 7.4 Hz, H-1′), 4.14 (2H, dd, *J* = 5.3, 3.3 Hz, H-3), 3.98 (1H, m, H-2), 3.82-3.91 (5H, m, H-1β, 4′, 2″, 4″, 6′β), 3.69-3.79 (5H, m, H-5′, 5″, 3″, 6″), 3.65 (2H, dd, *J* = 10.5, 4.6 Hz, H-1α, 6α), 3.54 (1H, m, H-2′), 3.50 (1H, m, H-3′), 2.77 (2H, t, *J* = 6.5 Hz, H-11‴), 2.35 (2H, t, *J*=7.5 Hz, H-2‴), 2.06 (4H, m, H-8‴, 14‴), 1.61 (2H, m, H-3‴), 1.28-1.37 (14H, m, H-4‴, 5‴, 6‴, 7‴, 8‴, 15‴, 16‴, 17‴), 0.90 (3H, t, *J*=7.0 Hz, H-18‴); ^{13}C NMR (126 MHz, Methanol-*d*$_4$) *δ*175.5 (s, C-1‴), 130.9 (d, C-9‴), 130.9 (s, C-13‴), 129.1 (d, C-10‴), 129.0 (d, C-12‴), 105.3 (d, C-1′), 100.5 (d, C-1″), 74.7 (d, C-3′), 74.6 (d, C-5′),

		72.6 (d, C-2′), 72.5 (d, C-5″), 72.1 (t, C-1), 71.4 (d, C-3″), 71.0 (d, C-4″), 70.2 (d, C-2), 70.1 (d, C-4′), 69.7 (d, C-2″), 67.8 (t, C-6′), 66.6 (t, C-3), 62.7 (t, C-6″), 34.9 (t, C-2‴), 32.7 (t, C-16‴), 30.7 (t, C-15‴), 30.5 (t, C-6‴), 30.3 (t, C-4‴), 30.2 (t, C-7‴), 30.2 (t, C-5‴), 28.2 (t, C-8‴), 28.2 (t, C-14‴), 26.5 (d, C-11‴), 26.0 (t, C-3‴), 23.6 (t, C-17‴), 14.4 (q, C-18‴);
3-*O*-octadeca-9*Z*,12*Z*,15*Z*-trienoylglyceryl-6'-*O*-(*α*-*D*-galactopyranosyl)-*β*-*D*-galactopyranoside (**6**)	[3]	$C_{33}H_{56}O_{14}Na$ (HR-ESI-MS *m/z* 699.38301); ^{1}H NMR (500 MHz, CD_3OD) δ_H 5.28-5.38 (6H, m, H-9‴, 10‴, 12‴, 13‴, 15‴, 16‴), 4.86 (1H, br s, H-1″), 4.24 (1H, d, *J* = 7.4 Hz, H-1′), 4.10 (2H, m, H-3), 3.98 (1H, dt, *J* = 10.1,5.0 Hz, H-6′β), 3.82-3.90 (5H, m, H-2, 2″, 4″, 4′, 1β), 3.77 (1H, dd, *J* = 10.1, 3.6 Hz, H-5″), 3.63-3.74 (6H, m, H-1, 3″, 6″, 5′, 6′α), 3.53 (1H, dd, *J* = 9.7, 7.3 Hz, H-2′), 3.48 (1H, dd, *J* = 9.7, 3.3 Hz, H-3′), 2.80 (2H, t, *J* =6.1 Hz, H-14‴), 2.77 (2H, t, *J* =6.4 Hz, H-11‴), 2.35 (2H, t, *J* =7.5 Hz, H-2‴), 2.06 (4H, m, H-8‴, 17‴), 1.61 (2H, m, H-3‴), 1.28-1.38 (8H, m, H-4‴～7‴), 0.96 (3H, t, *J*=7.5 Hz, H-8‴); ^{13}C NMR (126 MHz, CD_3OD) δ_C175.5 (s, C-1‴), 132.8 (d, C-16‴), 131.1 (d, C-9‴), 128.8 (d, C-10‴), 129.2 (d, C- 12‴), 129.1 (d, C-13‴), 128.2 (d, C-15‴), 105.3 (d, C-1′), 100.5 (d, C-1″), 74.7 (d, C-3′), 74.6 (d, C-5′), 72.6 (d, C-5″), 72.5 (d, C-2′), 72.1 (t, C-1), 71.5 (d, C-3″), 71.0 (d, C-4″), 70.2 (d, C-2), 70.1 (d, C-4′), 69.7 (d, C-2″), 67.8 (t, C-6′), 66.6 (t, C-3), 62.7 (t, C-6″), 34.9 (t, C-2‴), 30.7-30.2 (t, C-4‴～7‴), 28.2 (t, C-8‴), 26.5 (t, C-14‴), 26.4 (t, C-11‴), 26.0 (t, C-3‴), 21.5 (t, C-17‴), 14.7 (q, C-18‴);
1-*O*-palmitoyl-2-*O*-linoleoyl-3-*O*-[*α*-*D*-galactopyranosyl-(1→6)-*β*-*D*-galactopyranosyl]-glycerol (**7**)	[4]	$C_{49}H_{88}O_{15}Na$ (HR-ESI-MS *m/z* 939.59985 $[M + Na]^+$); ^{1}H NMR (500 MHz, CD_3OD) δ_H 5.32-5.34 (4H, m, H-9‴, 10‴, 12‴, 13‴), 5.24 (1H, m, H-2), 4.86 (1H, br s, H-1″), 4.43 (1H, dd, *J* = 12.1, 2.9 Hz, H-1β), 4.23 (1H, d, *J* = 7.2 Hz, H-1′), 4.21 (1H, dd, *J* = 12.1, 6.8 Hz, H-1α), 3.93 (1H, dd, *J* = 10.9, 5.4 Hz, H-3β), 3.82-3.89 (4H, overlap, H-6′β, 4″, 4′, 3α), 3.78 (1H, dd, *J* = 10.1, 3.7 Hz, H-2″), 3.69-3.74 (5H, overlap, H-5′, 3″, 5″, 6″), 3.66 (1H, dd, *J* = 9.9, 6.2 Hz, H-6′α), 3.50 (1H, dd, *J* = 9.7, 7.1 Hz, H-2′), 3.45 (1H, dd, *J* = 9.7, 3.1 Hz, H-3′), 2.77 (2H, t, *J* =6.5 Hz, H-11‴), 2.32 (2H, t, *J* =7.1 Hz, H-2‴), 2.31 (2H, t, *J* =7.3 Hz, H-2⁗), 2.06 (4H, m, H-8‴, 14‴), 1.59 (4H, m, H-3‴, 3⁗), 1.28-1.36 (38H, m, H-4⁗～15⁗, 4‴～7‴, 15‴～17‴), 0.90 (6H, t, *J* = 6.9 Hz, H-18‴, 16⁗); ^{13}C NMR (126 MHz, CD_3OD) δ_C175.0 (s, C-1‴), 174.7 (C-1⁗), 105.3 (d, C-1′), 100.6 (d, C-1″), 74.7 (d, C-3′), 74.6 (d, C-5′), 72.5 (d, C-2′), 72.4 (d, C-2), 71.7 (d, C-5″), 71.5 (d, C-2″), 71.1 (d, C-4″), 70.2 (d, C-3″), 70.1 (d, C-4′), 68.7 (t, C-3), 67.8 (t, C-6′), 64.0 (t, C-1), 62.8 (t, C-6″), 35.1 (t, C-3‴), 35.0 (t, C-3⁗), 33.1 (t, C-14⁗), 32.7 (t, C-16‴), 30.8-30.2 (t, C-4⁗～13⁗, 4‴～7‴, 15‴), 28.2 (t, C-8‴, 14‴), 26.6 (t, C-11‴), 26.0 (t, C-3‴, 3⁗), 23.7 (t, C-15⁗), 23.6 (t, C-17‴), 14.5 (q, C-18‴, 16⁗)
1-*O*-octadecanoyl-2-*O*-(9*Z*,12*Z*-octadecadienoyl)-3-*O*-[*α*-*D*-galactopyranosyl-	[5]	$C_{51}H_{92}O_{15}Na$ (HR-ESI-MS *m/z* 967.59784 $[M + Na]^+$); ^{1}H NMR (500 MHz, CD_3OD) δ_H 5.32-5.34 (4H, m, H-9⁗, 10⁗, 12⁗, 13⁗), 5.24 (1H, m, H-2), 4.86 (1H, br s, H-1″),

(1"→6')-*O*-β-*D*-galactopyranosyl] glycerol (**8**)		4.43 (1H, dd, *J* = 12.1, 2.9 Hz, H-1β), 4.23 (1H, d, *J* = 7.2 Hz, H-1′), 4.22 (1H, dd, *J* = 12.1, 6.7 Hz, H-1α), 3.93 (1H, dd, *J* = 10.9, 5.4 Hz, H-3β), 3.83-3.89 (3H, overlap, H-6′β, 4″, 4′), 3.77 (1H, dd, *J* = 10.0, 3.7 Hz, H-2″), 3.69-3.74 (6H, overlap, H-3α, 5′, 3″, 5″, 6″), 3.66 (1H, dd, *J* = 10.0, 6.2 Hz, H-6′α), 3.50 (1H, dd, *J* = 9.7, 7.1 Hz, H-2′), 3.45 (1H, dd, *J* = 9.7, 3.1 Hz, H-3′), 2.77 (2H, t, *J* =6.5 Hz, H-11″″), 2.32 (2H, t, *J* =7.2 Hz, H-2″″), 2.31 (2H, t, *J* =7.3 Hz, H-2‴), 2.05 (4H, m, H-8″″, 14″″), 1.59 (4H, m, H-3‴, 3″″), 1.28-1.36 (42H, m, H-4‴～17‴, 4″″～7″″, 15″″～17″″), 0.90 (6H, t, *J* = 6.9 Hz, H-18‴, 18″″); ^{13}C NMR (126 MHz, CD_3OD) δ_C175.0 (s, C-1‴),174.7 (C-1′), 105.3 (d, C-1′), 100.6 (d, C-1″), 74.7 (d, C-3′), 74.6 (d, C-5′), 72.5 (d, C-2′), 72.4 (d, C-2), 71.7 (d, C-5″), 71.5 (d, C-2″), 71.1 (d, C-4″), 70.2 (d, C-3″), 70.1 (d, C-4′), 68.7 (t, C-3), 67.8 (t, C-6′), 64.0 (t, C-1), 62.8 (t, C-6″), 35.1 (t, C-3‴), 35.0 (t, C-3″″), 33.1 (t, C-16″″), 32.7 (t, C-16‴), 30.8-30.2 (t, C-4‴～15‴, 4″″～7″″, 15″″), 28.2 (t, C-8″″, 14″″), 26.6 (t, C-11″″), 26.0 (t, C-3‴, 3″″), 23.7 (t, C-17″″), 23.6 (t, C-17‴), 14.5 (q, C-18‴, 18″″);
1-*O*-linoleoyl-2-*O*-oleoyl-3-*O*-[*α*-*D*-galactopyranosyl-(1→6)-*β*-*D*-galactopyranosyl]-glycerol (**9**)	[6]	$C_{51}H_{90}O_{15}Na$ (HR-ESI-MS *m/z* 965.61383 $[M + Na]^+$); ^{1}H NMR (500 MHz, CD_3OD) δ_H 5.30-5.34 (6H, m, H-9‴, 9″″, 10‴, 10″″, 12‴, 13‴), 5.24 (1H, m, H-2), 4.86 (1H, br s, H-1″), 4.42 (1H, dd, *J* = 12.1, 2.9 Hz, H-1β), 4.23 (1H, d, *J* = 7.1 Hz, H-1′), 4.21 (1H, dd, *J* = 12.1, 6.8 Hz, H-1α), 3.92 (1H, dd, *J* = 10.9, 5.4 Hz, H-3β), 3.83-3.88 (4H, overlap, H-6′β, 4″, 4′, 3α), 3.78 (1H, dd, *J* = 10.3, 3.7 Hz, H-2″), 3.69-3.75 (5H, overlap, H-5′, 3″, 5″, 6″), 3.66 (1H, dd, *J* = 10.0, 6.2 Hz, H-6′α), 3.51 (1H, dd, *J* = 9.7, 7.1 Hz, H-2′), 3.47 (1H, dd, *J* = 9.7, 3.2 Hz, H-3′), 2.77 (2H, t, *J* =6.4 Hz, H-11‴), 2.32 (2H, t, *J* =7.4 Hz, H-2‴), 2.31 (2H, t, *J* =7.4 Hz, H-2″″), 2.05 (8H, m, H-8‴, 8″″, 11″″, 14‴), 1.60 (4H, m, H-3‴, 3″″), 1.28-1.36 (34H, m, H-4″″～7″″, 12″″～17″″, 4‴～7‴, 15‴～17‴), 0.90 (6H, t, *J* = 7.0 Hz, H-18‴, 18″″); ^{13}C NMR (126 MHz, CD_3OD) δ_C175.0 (s, C-1‴), 174.7 (C-1″″), 131.0 (d, C-9‴, 9″″), 130.9 (s, C-10″″, 13‴), 129.1 (d, C-10‴), 129.0 (d, C-12‴), 105.3 (d, C-1′), 100.6 (d, C-1″), 74.7 (d, C-3′), 74.6 (d, C-5′), 72.5 (d, C-2′), 72.4 (d, C-2), 71.7 (d, C-5″), 71.5 (d, C-2″), 71.1 (d, C-4″), 70.2 (d, C-3″), 70.1 (d, C-4′), 68.7 (t, C-3), 67.8 (t, C-6′), 64.0 (t, C-1), 62.8 (t, C-6″), 35.1 (t, C-3‴), 35.0 (t, C-3″″), 33.1 (t, C-16″″), 32.7 (t, C-16‴), 30.8-30.2 (t, C-4″″～7″″, C-12″″～15″″, 4‴～7‴, 15‴), 28.2 (t, C-8‴, 8″″, 11″″, 14‴), 26.6 (t, C-11‴), 26.0 (t, C-3‴, 3″″), 23.7 (t, C-17″″), 23.6 (t, C-17‴), 14.5 (q, C-18‴, 18″″).
2,3-*O*-dioctadeca-9*Z*,12*Z*-dienoylglyceryl-6'-*O*-(*α*-*D*-galactopyranosyl)-*β*-*D*-galactopyranoside (**10**)	[3]	$C_{51}H_{88}O_{15}Na$ (HR-ESI-MS *m/z* 963.59937 $[M + Na]^+$); ^{1}H NMR (500 MHz, CD_3OD) δ_H 5.30-5.37 (8H, m, H-9‴, 9″″, 10‴, 10″″, 12‴, 12″″, 13‴, 13″″), 5.24 (1H, m, H-2), 4.86 (1H, br s, H-1″), 4.43 (1H, dd, *J* = 12.1, 2.9 Hz, H-3β), 4.23 (1H, d, *J* = 7.2 Hz, H-1′), 4.22 (1H, dd, *J* = 12.0, 6.6 Hz, H-3α), 3.93 (1H, dd, *J* = 10.9, 5.4 Hz, H-6′β), 3.81-3.91 (4H, overlap, H-1β, 2″, 4″, 5″), 3.78 (1H, dd, *J* = 10.1, 3.7 Hz, H-4′), 3.69-3.74 (5H, overlap, H-5′, 3″, 6′α, 6″), 3.67 (1H, dd, *J* = 10.0, 6.2 Hz, H-1α), 3.46-3.51 (2H, overlap,

		H-2′, 3′), 2.77 (4H, t, J = 6.5 Hz, H-11‴, 11⁗), 2.32 (2H, t, J = 7.1 Hz, H-2‴), 2.31 (2H, t, J = 7.2 Hz, H-2⁗), 2.06 (8H, m, H-8‴, 14‴, 8⁗, 14⁗), 1.60 (4H, m, H-3‴, 3⁗), 1.29-1.37 (28H, m, H-4‴～7‴, 15‴～17‴, 4⁗～7⁗, 15⁗～17⁗), 0.90 (6H, t, J = 6.8 Hz, H-18‴, 18⁗); ^{13}C NMR (126 MHz, CD_3OD) δ_C175.0 (s, C-1⁗), 174.7 (s, C-1‴), 131.0 (d, C-9‴, 9⁗), 130.9 (d, C-13‴, 13⁗), 129.1 (d, C-10‴, 10⁗), 129.0 (d, C-12‴, 12⁗), 105.3 (d, C-1′), 100.6 (d, C-1″), 74.7 (d, C-3′), 74.6 (d, C-5′), 72.5 (d, C-5″), 72.4 (d, C-2′), 71.7 (d, C-2), 71.5 (d, C-3″), 71.1 (d, C-4″), 70.2 (d, C-4′), 70.0 (d, C-2″), 68.7 (t, C-6′), 67.8 (t, C-1), 64.0 (t, C-3), 62.8 (t, C-6″), 35.1 (t, C-2⁗), 35.0 (t, C-2‴), 32.7 (t, C-16‴, 16⁗), 30.8-30.2 (t, C-4‴～7‴, 15‴, 4⁗～7⁗, 15⁗), 28.2 (t, C-8‴, 8⁗, 14‴, 14⁗), 26.6 (t, C-11‴, 11⁗), 26.0 (t, C-3‴, 3⁗), 23.6 (t, C-17‴, 17⁗), 14.5 (q, C-18‴, 18⁗);
2-*O*-octadeca-9*Z*,12*Z*-dienoyl-9*Z*,12*Z*,15*Z*-trienoylglyceryl-6'-*O*-(*α*-*D*-galactopyranosyl)-*β*-*D*-galactopyranoside (**11**)	[7]	$C_{51}H_{86}O_{15}Na$ (HR-ESI-MS m/z 961.58301 $[M + Na]^+$); ^{1}H NMR (500 MHz, CD_3OD) δ_H 5.30-5.38 (10H, m, H-9‴, 9⁗, 10‴, 10⁗, 12‴, 12⁗, 13‴, 13⁗, 15⁗, 16⁗), 5.24 (1H, m, H-2), 4.86 (1H, br s, H-1″), 4.43 (1H, dd, J = 12.4, 2.9 Hz, H-3β), 4.23 (1H, d, J = 7.2 Hz, H-1′), 4.22 (1H, dd, J = 12.1, 6.7 Hz, H-3α), 3.93 (1H, dd, J = 10.9, 5.4 Hz, H-6′β), 3.81-3.90 (4H, m, H-1β, 2″, 4″, 5″), 3.77 (1H, dd, J = 10.1, 3.7 Hz, H-4′), 3.69-3.74 (5H, m, H-5′, 3″, 6′α, 6″), 3.66 (1H, dd, J = 10.0, 6.2 Hz, H-1α), 3.46-3.51 (2H, dd, J = 8.5, 5.1 Hz, H-2′, 3′), 2.80 (2H, t, J = 6.2 Hz, 14⁗), 2.77 (4H, t, J = 6.5 Hz, H-11‴, 11⁗), 2.32 (2H, t, J = 7.1 Hz, H-2‴), 2.31 (2H, t, J = 7.2 Hz, H-2⁗), 2.08 (2H, m, H-17⁗), 2.06 (6H, m, H-8‴, 14‴, 8⁗), 1.60 (4H, m, H-3‴, 3⁗), 1.28-1.38 (22H, m, H-4‴～7‴, 15‴～17‴, 4⁗～7⁗), 0.97 (3H, t, J=7.5 Hz, H-18⁗), 0.90 (3H, t, J=7.0 Hz, H-18‴); ^{13}C NMR (126 MHz, CD_3OD) δ_C175.0 (s, C-1⁗), 174.7 (s, C-1‴), 132.7 (d, C-16⁗), 131.0 (d, C-9⁗), 130.8 (d, C-9‴, 13‴), 129.1 (d, C-10⁗), 129.0 (d, C-10‴, 12‴), 129.0 (d, C-13⁗), 128.9 (d, C-12⁗), 128.2 (d, C-15⁗), 105.3 (d, C-1′), 100.6 (d, C-1″), 74.7 (d, C-3′), 74.6 (d, C-5′), 72.5 (d, C-5″), 72.4 (d, C-2′), 71.7 (d, C-2), 71.5 (d, C-3″), 71.1 (d, C-4″), 70.2 (d, C-4′), 70.1 (d, C-2″), 68.7 (t, C-6′), 67.8 (t, C-1), 64.0 (t, C-3), 62.8 (t, C-6″), 35.1 (t, C-2⁗), 35.0 (t, C-2‴), 32.7 (t, C-16‴), 30.7-30.2 (t, C-4‴～7‴, 15‴, 4⁗～7⁗), 28.2 (t, C-8‴, 8⁗, 14‴), 26.6 (t, C-11‴, 11⁗), 26.4 (t, C-14⁗), 26.0 (t, C-3‴, 3⁗), 23.6 (t, C-17‴), 21.5 (t, C-17⁗), 14.7 (q, C-18⁗), 14.5 (q, C-18‴)
1-*O*-(9*Z*,12*Z*-octadecadienoly)-3-*O*-*β*-galactopyranosylglycerol (**12**)	[8]	$C_{27}H_{48}O_9Na$ (HRESIMS m/z 539.31982 $[M + Na]^+$); ^{1}H NMR (500 MHz, CD_3OD) δ5.33 (4H, m, H-9″, 10″, 12″, 13″), 4.22 (1H, d, J = 7.6 Hz, H-1′), 4.10-4.17 (2H, m, H-1), 3.98 (1H, m, H-2), 3.91 (1H, dd, J = 10.5, 5.1 Hz, H-3b), 3.81 (1H, dd, J = 3.3, 0.8 Hz, H-4′), 3.73 (2H, dd, J = 9.7, 6.1 Hz, H-6′), 3.64 (H, dd, J = 9.7, 6.1 Hz, H-3a), 3.53 (H, dd, J = 8.6, 6.4 Hz, H-2′), 3.50 (1H, m, H-5′), 3.46 (1H, dd, J = 9.7, 3.4 Hz, H-3′), 2.77 (2H, t, J = 6.5 Hz, H-11″), 2.35 (2H, t, J = 7.5 Hz, H-2″), 2.06 (4H, m, H-8″, 14″), 1.61 (2H, m, H-3″), 1.28-1.37 (14H, m, H-4″-7″, 15″-17″), 0.90 (3H, t, J = 7.0 Hz, H-18″);

		^{13}C NMR (126 MHz, CD_3OD) δ175.4 (s, C-1″), 130.9 (d, C-9″), 130.9 (s, C-13″), 129.1 (d, C-10″), 129.0 (d, C-12″), 105.3 (d, C-1′), 76.8 (d, C-5′), 74.8 (d, C-3′), 72.6 (d, C-2′), 71.9 (t, C-3), 70.3 (d, C-4′), 69.8 (d, C-2), 66.6 (t, C-1), 62.5 (t, C-6′), 34.9 (t, C-2″), 32.7 (t, C-16″), 30.2-30.7 (t, C-4″,5″,6″,7″,15″,16″), 28.2 (t, C-8″), 28.2 (t, C-14″), 26.5 (t, C-11″), 26.0 (t, C-3″), 23.6 (t, C-17″), 14.4 (q, C-18″);
3-*O*-octadeca-9*Z*,12*Z*,15*Z*-trienoylglyceryl-*O*-*β*-*D*-galactopyranoside (**13**)	[1]	$C_{27}H_{46}O_9K$ (HRESIMS *m/z* 553.45825 [*M* + K]$^+$); ^{1}H NMR (500 MHz, Methanol-*d*$_4$) δ5.30-5.36 (6H, m, H-9″, 10″, 12″, 13″, 15″, 16″), 4.30 (1H, d, *J*=7.5 Hz, H-1′), 4.03 (2H, m, H-3), 3.93 (1H, m, H-2), 3.82 (1H, dd, *J*=10.0, 4.3 Hz, H-1a), 3.69 (2H, m, H-6′), 3.75 (1H, m, H-4′), 3.69 (1H, dd, *J*=10.0, 5.2 Hz, H-1b), 3.49-3.52 (2H, m, H-2′, 5′), 3.43 (1H, dd, *J*=9.7, 3.3 Hz H-3′), 2.77 (4H, t, *J*=6.5 Hz, H-11″, 14″), 2.28 (2H, t, *J*=7.2 Hz, H-2″), 2.06 (2H, m, H-8″), 2.03 (2H, m, H-17″), 1.60 (2H, m, H-3″), 1.27-1.34 (8H, m, H-4″-7″), 0.89 (3H, t, *J*=6.8 Hz, H-18″); ^{13}C NMR (126 MHz, Methanol-*d*$_4$) δ175.2 (s, C-1″), 131.9 (d, C-16″), 131.2 (d, C-15″), 129.2 (d, C-13″), 129.1 (d, C-12″), 128.7 (d, C-10″), 128.2 (d, C-9″), 105.3 (d, C-1′), 76.7 (d, C-5′), 74.8 (d, C-3′), 72.6 (d, C-2′), 71.9 (t, C-1), 70.2 (d, C-4′), 69.8 (d, C-2), 66.6 (t, C-3), 62.6 (t, C-6′), 35.0 (t, C-2″), 30.2-30.8 (t, C-4″, 5″,6″, 7″), 28.2 (t, C-8″), 26.7 (t, C-14″), 26.6 (t, C-11″), 26.0 (t, C-3″), 19.8 (t, C-17″), 14.5 (q, C-18″);
1-*O*-oleoyl-2-*O*-myristoyl-glyceryl-*O*-*β*-*D*-galactopyranoside (**14**)	[3]	$C_{39}H_{74}O_{10}Na$ (HRESIMS *m/z* 725.44360 [*M* + Na]$^+$) ^{1}H NMR (500 MHz, $CDCl_3$) δ5.26 (1H, m, H-2), 4.39 (1H, dd, *J* = 12.0, 3.3 Hz, H-1a), 4.27 (1H, d, *J* = 7.5 Hz, H-1′), 4.21 (1H, dd, *J* = 12.0, 6.5 Hz, H-1b), 3.96 (1H, dd, *J* = 11.9, 5.9 Hz, H-3a), 3.91 (1H, dd, *J* = 11.9, 5.9 Hz, H-6′a), 3.84 (1H, br d, *J* = 4.1 Hz, H-4′), 3.73 (1H, dd, *J* = 11.2, 5.3 Hz, H-3b), 3.66 (1H, dd, *J* = 11.5, 5.4 Hz, H-6′b), 3.51 (1H, overlap, H-2′), 3.59 (1H, dd, *J* = 9.5, 3.2 Hz, H-5′), 3.54 (1H, dd, *J* = 9.7, 3.3 Hz, H-3′) 2.32 (2H, t, *J* = 7.6 Hz, H-2″), 2.30 (2H, t, *J* = 7.7 Hz, H-2‴), 1.60 (4H, m, H-3″, 3‴), 1.29-1.37 (44H, m, H-4″～13″, 4‴～15‴), 0.89 (6H, t, *J* = 6.9 Hz, H-14″, 16‴); ^{13}C NMR (126 MHz, $CDCl_3$) δ173.8 (s, C-1″), 173.5 (s, C-1‴), 104.0 (d, C-1′), 76.8 (d, C-5′), 74.5 (d, C-3′), 73.5 (d, C-2′), 71.6 (d, C-2), 70.2 (d, C-4′), 69.5 (t, C-3), 68.4 (t, C-1), 62.8 (t, C-6′), 34.3 (t, C-2″), 34.1 (t, C-2‴), 31.5 (t, C-12″, 14‴), 29.2-29.6 (t, C-4″～11″, 4‴～13‴), 22.6 (t, C-13″, 15‴), 14.1 (q, C-14″, 16‴);
1,2-*O*-diacyl-3-*O*-*β*-*D*-galactopyranosyl glycerols (**15**)	[5]	$C_{43}H_{78}O_{10}Na$ (HRESIMS *m/z* 777.54822 [*M* + Na]$^+$); ^{1}H NMR (500 MHz, CD_3OD) δ5.35 (4H, m, H-9″, 10″, 12″, 13″), 5.26 (1H, m, H-2), 4.44 (1H, dd, *J* = 12.1, 3.0 Hz, H-1a), 4.22 (1H, d, *J* = 7.6 Hz, H-1′), 4.21 (1H, dd, *J* = 12.0, 6.8 Hz, H-1b), 3.98 (1H, dd, *J* = 10.9, 5.4 Hz, H-3a), 3.82 (1H, dd, *J* = 3.2, 0.7 Hz, H-4′), 3.76 (1H, dd, *J* = 11.5, 6.9 Hz, H-6′a), 3.74 (1H, dd, *J* = 10.9, 5.4 Hz, H-3b), 3.72 (1H, dd, *J* = 11.5, 5.4 Hz, H-6′b), 3.51 (1H, overlap, H-2′), 3.49 (1H, overlap, H-5′), 3.45 (1H, dd, *J* = 9.7, 3.3 Hz, H-3′) , 2.77 (2H, t, *J* = 6.5 Hz, H-11″), 2.33 (2H, t, *J* = 7.4 Hz, H-2″), 2.30 (2H, t, *J* = 7.4

		Hz, H-2‴), 2.06 (4H, m, H-8″, 14″), 1.61 (4H, m, H-3″, 3‴), 1.28-1.37 (38H, m, H-4″～7″, 15″～17″, 4‴～15‴), 0.90 (6H, t, *J* = 7.0 Hz, H-18″, 18‴); ^{13}C NMR (126 MHz, CD_3OD) *δ*175.0 (s, C-1″), 174.7 (s, C-1‴), 131.0 (d, C-9″), 130.8 (s, C-13″), 129.1 (d, C-10″), 129.0 (d, C-12″), 105.4 (d, C-1′), 76.8 (d, C-5′), 74.9 (d, C-3′), 72.4 (d, C-2′), 71.8 (d, C-2), 70.2 (d, C-4′), 68.7 (t, C-3), 64.0 (t, C-1), 62.5 (t, C-6′), 35.1 (t, C-2″), 35.0 (t, C-2‴), 32.7 (t, C-16″, 14‴), 30.2-30.8 (t, C-4″～7″, 15″, 4‴～13‴), 28.2 (t, C-8″, 14″), 26.6 (t, C-11″), 26.0 (t, C-3″, 3‴), 23.8 (t, C-17″), 23.7 (t, C-15‴), 14.5 (q, C-18″, 16‴);
2,3-*O*-dioctadeca-9*Z*,12*Z*-dienoylglyceryl-*O*-*β*-*D*-galactopyranoside (**16**)	[3]	$C_{45}H_{78}O_{10}Na$ (HRESIMS *m/z* 801.54785 [*M* + Na]$^+$); ^{1}H NMR (500 MHz, $CDCl_3$) δ_H 5.28-5.39 (9H, m, H-9″, 9‴, 10″, 10‴, 12″, 12‴, 13″, 13‴, H-2), 4.39 (1H, dd, *J* = 12.0, 3.3 Hz, H-3β), 4.27 (1H, d, *J* = 7.5 Hz, H-1′), 4.21 (1H, dd, *J* = 12.0, 6.5 Hz, H-3α), 3.96 (1H, dd, *J* = 11.9, 5.9 Hz, H-1β), 3.91 (1H, dd, *J* = 11.2, 5.4 Hz, H-4′), 3.73 (1H, dd, *J* = 11.2, 6.4 Hz, H-1α), 3.66 (1H, m, H-6′β), 3.59 (1H, dd, *J* = 9.5, 3.2 Hz, H-6′α), 3.54 (1H, t, *J* = 5.1 Hz, H-2′), 3.27 (2H, m, H-3′, 5′), 2.76 (4H, t, *J* = 6.7 Hz, H-11″, 11‴), 2.32 (2H, t, *J* = 7.6 Hz, H-2″), 2.31 (2H, t, *J* = 7.7 Hz, H-2‴), 2.06 (8H, m, H-8″, 14″, 8‴, 14‴), 1.61 (4H, m, H-3″, 3‴), 1.26-1.37 (28H, m, H-4″～7″, 15″～17″, 4‴～7‴, 15‴～17‴), 0.90 (6H, t, *J* = 6.7 Hz, H-18″, 18‴); ^{13}C NMR (126 MHz, $CDCl_3$) *δ*173.8 (s, C-1″), 173.5 (s, C-1‴), 130.3 (d, C-9‴), 130.0 (s, C-13‴), 128.1 (d, C-10‴), 127.9 (d, C-12‴), 104.0 (d, C-1′), 76.8 (d, C-5′), 74.5 (d, C-3′), 73.5 (d, C-2′), 71.6 (d, C-2), 70.2 (t, C-4′), 69.5 (d, C-3), 68.4 (d, C-1), 62.8 (t, C-6′), 34.3 (t, C-2‴), 34.1 (t, C-2″), 31.5 (t, C-16″, 16‴), 29.6-29.1 (t, C-4″～7″, 15″, 4‴～7‴, 15‴), 27.2 (t, C-8″, 8‴, 14″, 14‴), 25.6 (t, C-11″, 11‴), 24.9 (t, C-3″, 3‴), 22.6 (t, C-17″, 17‴), 14.1(q, C-18″, 18‴);
1-*O*-(9*Z*, 12*Z*-octadecadienoyl)-3-*O*-[*β*-*D*-galactopyranosyl-(1→6)-*O*-*β*-*D*-galactopyranosyl-(1→6)-*O*-*β*-*D*-galactopyranosyl] glycerol (**17**)	[9]	$C_{39}H_{68}O_{19}Na$ (HRESIMS *m/z* 863.42169 [*M* + Na]$^+$); ^{1}H NMR (500 MHz, CD_3OD) δ_H 5.28-5.38 (4H, m, H-9′, 10′, 12′, 13′), 4.85 (1H, br s, H-1⁗), 4.30 (1H, d, *J* = 7.5 Hz, H-1‴), 4.27 (1H, d, *J* = 7.6 Hz, H-1″), 4.16 (1H, dd, *J* = 11.4, 4.4 Hz, H-1β), 4.13 (1H, dd, *J* = 11.4, 6.3 Hz, H-1α), 4.06 (1H, t, *J* = 6.1 Hz, H-3β), 3.94-4.01 (4H, m, H-2, 6″β, 6‴β, 4⁗), 3.85-3.90 (2H, m, H-2⁗, 6‴α), 3.80-3.82 (2H, m, H-4‴, 5⁗), 3.78 (1H, dd, *J* = 7.2, 3.3 Hz, H-6⁗β), 3.69-3.78 (6H, m, H-6⁗α, 4‴, 5‴, 5⁗, 3α, 3⁗), 3.66 (1H, dd, *J* = 10.6, 5.0 Hz, H-6″α), 3.50-3.53 (3H, m, H-2″, 2⁗, 3⁗), 3.46 (1H, dd, *J*=9.7, 3.3 Hz, H-3″), 2.77 (2H, t, *J* = 6.5 Hz, H-11′), 2.35 (2H, t, *J* = 7.5 Hz, H-2′), 2.06 (4H, m, H-8′, 14′), 1.61 (2H, m, H-3′), 1.28-1.36 (14H, m, H-4′～7′, 15′～17′), 0.90 (3H, t, *J* = 6.9 Hz, H-18′); ^{13}C NMR (126 MHz, CD_3OD) δ_C175.6 (s, C-1′), 130.9 (d, C-9′, 13′), 129.1 (d, C-10′), 129.0 (d, C-12′), 105.3 (d, C-1′), 105.3 (d, C-1″), 100.8 (d, C-1‴), 76.7 (d, C-5″), 74.9 (d, C-3‴), 74.6 (d, C-3″), 74.5 (d, C-5‴), 72.6 (d, C-2″, 2‴), 72.1 (t, C-3), 71.4 (d, C-3⁗), 71.2 (d, C-4⁗), 70.8 (d, C-5⁗), 70.3 (d, C-4‴), 70.2 (d, C-2⁗), 70.1 (d, C-4″), 69.7 (t, C-6″), 69.6 (d, C-2), 68.1 (t, C-6‴), 66.7 (t, C-1), 62.5 (t, C-6⁗), 34.9 (t, C-

		2′), 32.7 (t, C-16′), 30.7-30.2 (t, C-4′～7′, 15′), 28.2 (t, C-8′,14′), 26.6 (t, C-11′), 26.0 (t, C-3′), 23.7 (t, C-17′), 14.5 (q, C-18′);
1,2-*O*-(9*Z*,12*Z*-octadecadienoyl)-3-*O*-[*α*-*D*-galactopyranosyl-(1'''''→6'''')-*O*-*β*-*D*-galactopyranosyl-(1''''→6''')-*O*-*β*-*D*-galactopyranosyl]-glycerol (**18**)	[10]	$C_{57}H_{98}O_{20}Na$ (HRESIMS *m/z* 1125.65271 $[M + Na]^+$); ^{1}H NMR (500 MHz, CD_3OD) δ_H 5.29-5.38 (8H, m, H-9′, 9″, 10′, 10″, 12′, 12″, 13′, 13″), 5.26 (1H, m, H-2), 4.86 (1H, br s, H-1″‴), 4.43 (1H, dd, *J* = 12.1, 2.8 Hz, H-1β), 4.30 (1H, d, *J* = 7.5 Hz, H-1⁗), 4.27 (1H, d, *J* = 7.6 Hz, H-1‴), 4.23 (1H, dd, *J* = 12.1, 6.9 Hz, H-1α), 4.04 (1H, t, *J* = 6.6 Hz, H-3β), 3.93-3.99 (3H, m, 6‴β, 6⁗β, 4″‴), 3.86-3.89 (2H, m, H-2″‴, 6⁗α), 3.81-3.83 (2H, m, H-4⁗, 5″‴), 3.69-3.78 (7H, m, H-6″‴, 4‴, 5‴, 5⁗, 3α, 3⁗), 3.49-3.53 (4H, m, H-2‴, 2⁗, 3″‴, 6‴α), 3.46 (1H, dd, *J* = 9.7, 3.3 Hz, H-3‴), 2.77 (4H, t, *J* = 6.6 Hz, H-11′, 11″), 2.33 (2H, t, *J* = 7.9 Hz, H-2′), 2.31 (2H, t, *J* = 7.8 Hz, H-2″), 2.06 (8H, m, H-8′, 8″, 14′, 14″), 1.60 (4H, m, H-3′, 3″), 1.29-1.37 (28H, m, H-4′～7′, 15′～17′, 4″～7″, 15″～17″), 0.90 (6H, t, *J* = 6.8 Hz, H-18′, 18″); ^{13}C NMR (126 MHz, CD_3OD) δ_C175.1 (s, C-1′), 174.8 (s, C-1″), 131.0 (d, C-9′, 9″), 130.9 (d, C-13′, 13″), 129.1 (d, C-10′, 10″, 12′, 12″), 105.3 (d, C-1⁗), 105.2 (d, C-1‴), 100.9 (d, C-1″‴), 76.7 (d, C-5‴), 74.9 (d, C-3⁗), 74.6 (d, C-3‴), 74.5 (d, C-5⁗), 72.5 (d, C-2⁗), 72.4 (d, C-2‴), 71.8 (d, C-2), 71.4 (d, C-3″‴), 71.2 (d, C-4″‴), 70.8 (d, C-5″‴), 70.3 (d, C-4⁗), 70.2 (d, C-2″‴), 70.1 (d, C-4‴), 69.8 (t, C-6‴), 68.8 (t, C-3), 68.3 (t, C-6⁗), 64.1 (t, C-1), 62.6 (t, C-6″‴), 35.2 (t, C-2′), 35.0 (t, C-2″), 32.7 (t, C-16′, 16″), 30.8-30.2 (t, C-4′～7′, 15′, 4″～7″, 15″), 28.2 (t, C-8′, 8″, 14′, 14″), 26.6 (t, C-11′, 11″), 26.0 (t, C-3′, 3″), 23.7 (t, C-17′, 17″), 14.5 (q, C-18′, 18″);

References

1. Suedee, A.; Tewtrakul, S.; Panichayupakaranant, P. Anti-HIV-1 integrase compound from *Pometia pinnata* leaves. *Pharm. Bio*l. **2013**, *51*, 1256-1261.
2. Mei, W.L.; Ni, W.; Liu, H.Y.; Chen, C.X. Studied on the constituents of Cinnamomum zeylanicum. *Nat. Prod. Res. Dev*. **2002**, *14*, 14-17.
3. Kiem, P.V.; Minh, C.V.; Nhiem, N.X.; Cuong, N.X.; Tai, B.H.; Quang, T.H.; Anh, H.L.T.; Yen, P.H.; Ban, N.K.; Kim, S.H.; Xin, M.J.; Cha, J.Y.; Lee, Y.M.; Kim, Y.H. Inhibitory Effect on TNF-α-Induced IL-8 Secretion in HT-29 Cell Line by Glyceroglycolipids from the Leaves of Ficus macrocarpa. *Arch Pharm. Res*. **2012**, *35*, 2135-2142.
4. Murakami, N.; Morimoto, T.; Imamura, H.; Ueda, T.; Nagai, S.I.; Sakakibara, J. Studies on Glycolipids III Glyceroglycolipids from an Axenically Cultured Cyanobacterium, Phormidium tenue. *Chem. Pharm. Bull*. **1991**, *39*, 2277-2281
5. Jung, J.H.; Lee, H.; Kang, S.S. Diacylglycerylgalactosides from *Arisaema amurense*. *Phytochemistry* **1996**, *42*, 447-452.

6. Bianco, A.; Mazzei, R.A.; Melchioni, C.; Scarpati, M.L.; Romeo, G.; Uccella, N. Microcomponents of olive oil. Part II: Digalactosyldiacylglycerols from *OZea europaea*. Food Chemistry **1998**, *62*, 343-346.
7. Chai, X.Y.; Bai, C.C.; Song, Y.L.; Chen, Y.P.; Li, F.F.; Tu, P.F. Chemical Constituents from the Leaves of Itoa orientalis. *Chin. J. Nat. Med*. **2008**, *6*, 179-182.
8. Kwon, H.C.; Zee, O.P.; Lee, K.R. Two New Monogalactosylacylglycerols from *Hydrocotyle ramiflora*. *Planta Med.* **1998**, *64*, 477-479.
9. Jiang, Z.G.; Du, Q.Z. Two new glyceroglycolipids from the fruits of *Cucurbita moschata*. *J. Chem. Res*. **2009**, 3, 157-159
10. Liu, L.P.; Zhang, J.Q.; Wang, X.Y.; Wang, H.B. Glycoglycerolipids from *Stellera chamaejasme*. *Nat. Prod. Commun.* **2012**, *7*, 1499-1500.

The UHPLC-ESI-MS Analysis of the Crude Ethyl Acetate Extract of PF02-2 and Oat Medium

In order to confirm the compounds (**1-18**) are isolated from *Tubeufia rubra* PF02-2 and not from oat medium, UHPLC-ESI-MS experiments were performed. A flask of 1 L containing 200 g oat and 150 mL distilled water was sterilized for 21 minutes at 121℃. Then the oat medium was extracted three times with EtOAc at room temperature for 48 h and the combined EtOAc extracts were concentrated under reduced pressure to give a oat crude extract. The samples which 3 mg ethyl acetate extract of PF02-2 and oat medium were dissolved in 6 mL methanol respectively were subjected to UHPLC-ESI-MS analysis. The retention time, MASS and MS/MS data of compounds (**1-18**) were detected from UHPLC-ESI-MS chromatograms of ethyl acetate extract of PF02-2 and oat medium, respectively. The results shown the fact that the retention time, MASS and MS/MS data of compounds (**1-18**) were just detected from the ethyl acetate extract of PF02-2, However, in the blank medium, the retention time, MASS and MS/MS data of compounds **1-18** were not detected. Taking compound **1** and compound **2** as examples, HR-ESI-MS ($[M + H]^+$ at m/z 983.62543 of compound **1** was detected (t_R=42.69 min) in the UHPLC-ESI-MS chromatogram of crude ethyl acetate extract of PF02-2 (Fig. S1, 3A, and 3B), while mass data of compound **1** was not detected in crude ethyl acetate extract of oat medium (Fig. S2, 3C, and 3D). Similarly, HR-ESI-MS ($[M + Na]^+$ at m/z 981.61023 of compound **2** was also detected (t_R=43.20 min) in UHPLC-ESI-MS chromatogram of crude ethyl acetate extract of PF02-2 (Fig. S1, 4A, and 4B), but mass data of compound **2** was not detected in crude ethyl acetate extract of oat medium (Fig. S2, 4C, and 4D). As a result, we confirm the compounds (**1-18**) are isolated from *Tubeufia rubra* PF02-2.

Chromatographic condition

Instrument: A Thermo Scientific Dionex Ultimate 3000 UHPLC system equipped with a Thermo high resolution Q Exactive focus mass spectrometer; Chromatographic column: Thermo Scientific HYPERSIL GOLD C18 (2.1×100 mm, 1.9 µm); Flow rate: 0.3 mL/min; Column temperature: 25 °C; Injection volume: 5 µL;

Table S1. Mobile phase for the UHPLC (A: H_2O; B: CH_3OH)

Time (min)	A %	B %
0.0	80	20
40	10	90
50	0	100
51	80	20
54	80	20

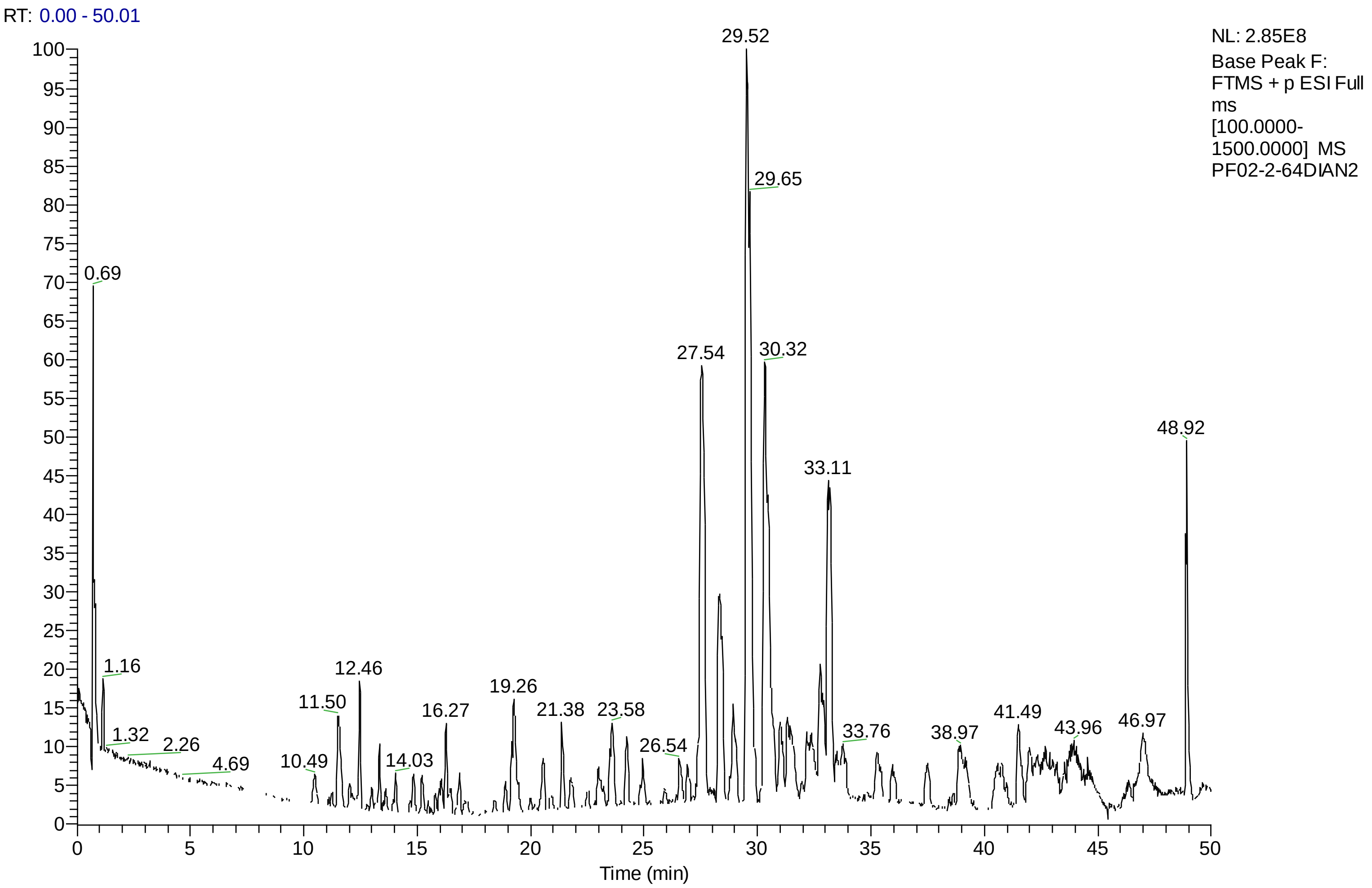

Figure S1 UHPLC-ESI-MS chromatogram of ethyl acetate crude extract of PF02-2

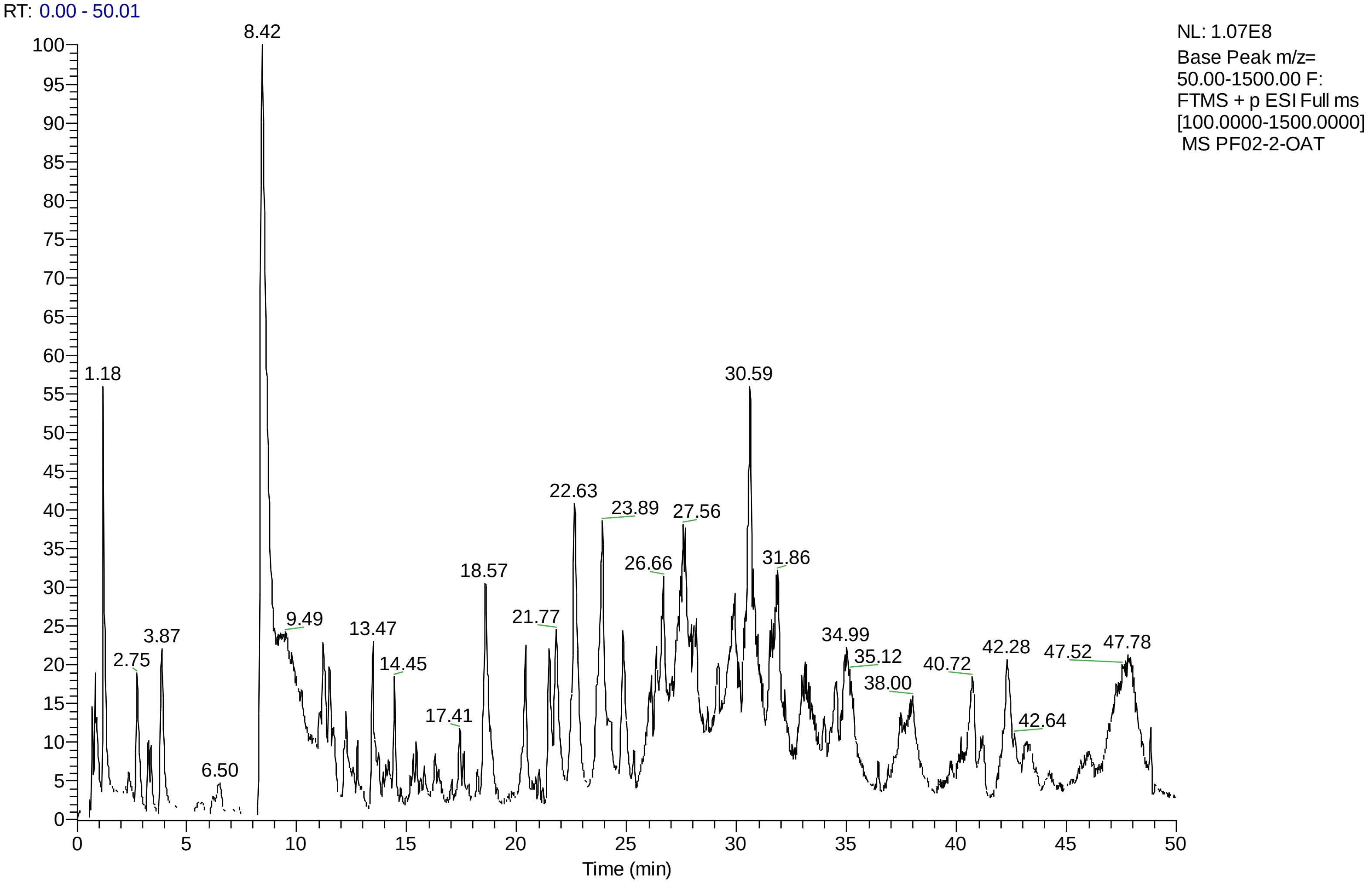

Figure S2 UHPLC-ESI-MS chromatogram of ethyl acetate crude extract of oat medium

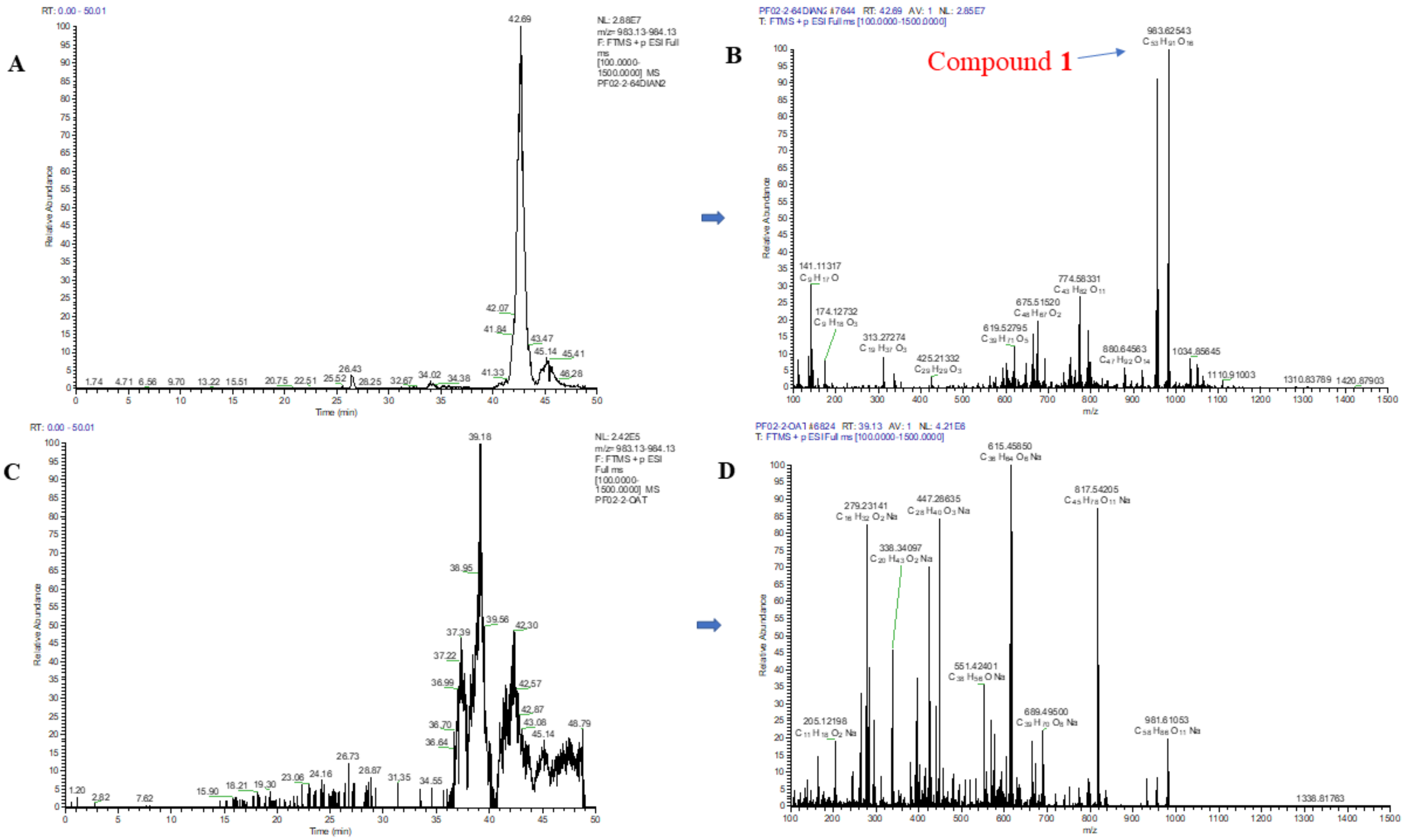

Figure S3 base peak chromatograms of **1** from the ethyl acetate crude extract PF02-2 and oat medium

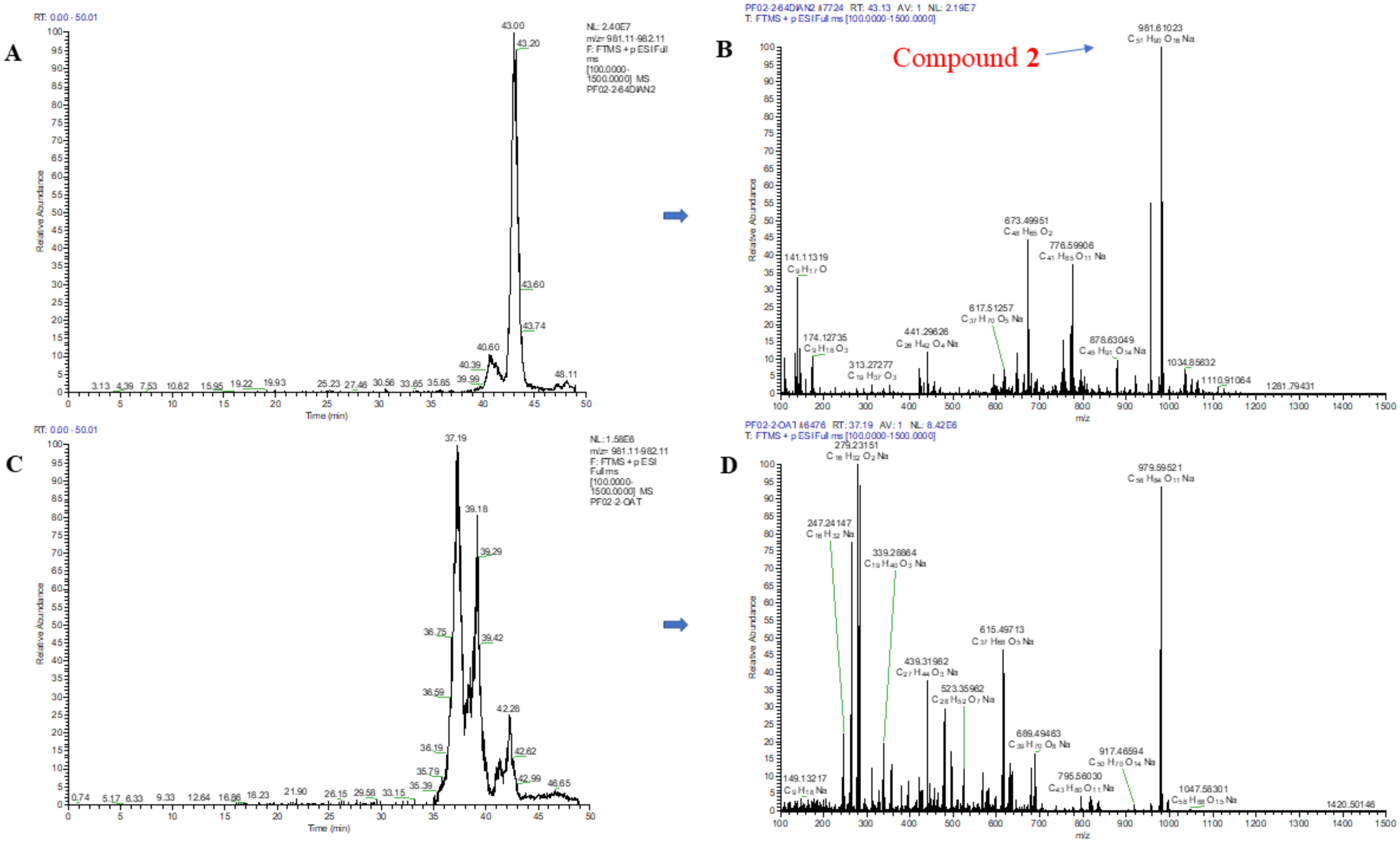

Figure S4 base peak chromatograms of **2** from the ethyl acetate crude extract PF02-2 and oat medium
